# Comparative diagnostic accuracy studies with an imperfect reference standard – a comparison of correction methods

**DOI:** 10.1186/s12874-021-01255-4

**Published:** 2021-04-12

**Authors:** Chinyereugo M. Umemneku Chikere, Kevin J. Wilson, A. Joy Allen, Luke Vale

**Affiliations:** 1 Population Health Science Institute, Faculty of Medical Sciences, Newcastle University, Newcastle upon Tyne, UK; 2 School of Mathematics, Statistics and Physics, Newcastle University, Newcastle upon Tyne, UK; 3 National Institute for Health Research, Newcastle In Vitro Diagnostics Co-operative, Newcastle University, Newcastle upon Tyne, UK

**Keywords:** Diagnostic accuracy, Imperfect reference standard, Sensitivity, Specificity, Staquet, Brenner, Correction method

## Abstract

**Background:**

Staquet et al. and Brenner both developed correction methods to estimate the sensitivity and specificity of a binary-response index test when the reference standard is imperfect and its sensitivity and specificity are known. However, to our knowledge, no study has compared the statistical properties of these methods, despite their long application in diagnostic accuracy studies.

**Aim:**

To compare the correction methods developed by Staquet et al. and Brenner.

**Methods:**

Simulations techniques were employed to compare the methods under assumptions that the new test and the reference standard are conditionally independent or dependent given the true disease status of an individual. Three clinical datasets were analysed to understand the impact of using each method to inform clinical decision-making.

**Results:**

Under the assumption of conditional independence, the Staquet et al. correction method outperforms the Brenner correction method irrespective of the prevalence of disease and whether the performance of the reference standard is better or worse than the index test. However, when the prevalence of the disease is high (> 0.9) or low (< 0.1), the Staquet et al. correction method can produce illogical results (i.e. results outside [0,1]). Under the assumption of conditional dependence; both methods failed to estimate the sensitivity and specificity of the index test especially when the covariance terms between the index test and the reference standard is not close to zero.

**Conclusion:**

When the new test and the imperfect reference standard are conditionally independent, and the sensitivity and specificity of the imperfect reference standard are known, the Staquet et al. correction method outperforms the Brenner method. However, where the prevalence of the target condition is very high or low or the two tests are conditionally dependent, other statistical methods such as latent class approaches should be considered.

**Supplementary Information:**

The online version contains supplementary material available at 10.1186/s12874-021-01255-4.

## Background

The diagnostic accuracy measures (sensitivity and specificity) of a new test are traditionally estimated through comparison with the best available reference standard. The reference standard is often assumed to be a *“gold standard*”, that is, “error free”. However, no test is perfect and ignoring this imperfection can result in either over or underestimating the accuracy of a new test (the index test) [1].

Following the reviews by Rutjes et al. [2] and Chikere et al. [3], three statistical methods (Gart and Buck [4], Staquet et al. [5], and Brenner [6]) were identified as being appropriate to evaluate the sensitivity and specificity of a binary response index test when the sensitivity and specificity of the imperfect reference standard are known and the index test and reference standard are conditionally independent. The estimates of the sensitivity and specificity of the imperfect reference standard can be obtained from previous validation studies, experimental or field studies. The three statistical methods are referred to as “*correction methods”*, because they aim to correct the estimated sensitivity and specificity of the index test using the available information (sensitivity and specificity) of the imperfect reference standard via algebraic functions. In addition, these correction methods do not require probabilistic modelling like latent class models [7, 8]. Both the correction methods and latent class models assume that the true disease status of the participants are unknown (latent). However, the latent class models are “*probabilistic*” approaches that estimate the accuracy measures of the index test and / or the reference standard via a statistical model. In addition, the Bayesian latent class models [9] incorporate other sources of information about the parameters of interest aside the from observation to make inference about the parameters of interest.

It is possible that there are certain scenarios where one correction method is more appropriate or may outperform the other. Hence, we decided to explore these correction methods to provide recommendations to test evaluators. To our knowledge, no study has directly compared these correction methods.

## Methods

### Notation

Let IT and RS denote index test and reference standard respectively. The results from both tests are considered to be binary (diseased and non-diseased). The results from the participants are often classified into a two-by-two contingency table (Table 1), which displays the number of participants with each combination of the test results. Notation used in this paper is reported in Table 2.

**Table 1:**
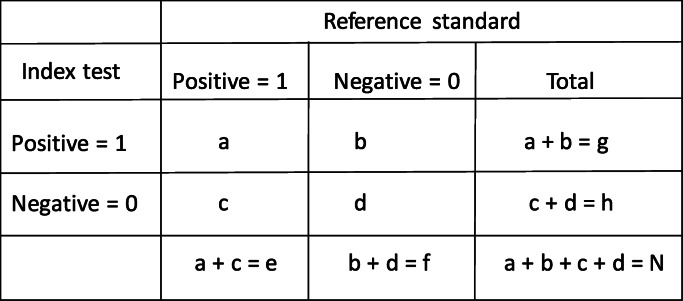
2 by 2 contingency table of the index test and imperfect reference standard

**Table 2:** Table of Notation

Notation	Meaning
*Sn*_*IT*_	Sensitivity of the index test
*Sp*_*IT*_	Specificity of the index test
*Sn*_*RS*_	Sensitivity of the reference standard
*Sp*_*RS*_	Specificity of the reference standard
*Sn*_*cor*_	Corrected sensitivity of index test
*Sp*_*cor*_	Corrected specificity of index test
$\hat{P}$	Estimated population prevalence
*J*	Youden’s index, *Sn*_*RS*_ + *Sp*_*RS*_ − 1
*Prr*	Sample prevalence

The classical estimates of the sensitivity and specificity of the index test, assuming that the reference standard is a gold standard, are:

1 $$ S{n}_{IT}=\frac{a}{e}\kern1.75em S{p}_{IT}=\frac{d}{f}\kern2em Prr=\frac{e}{N}\kern1.75em $$

### Correction methods

The correction methods identified from the systematic reviews [2, 3] were the Gart and Buck [4] correction method, the Staquet et al. [5] correction method and the Brenner [6] correction method.

#### Gart and Buck [4] correction method

The pair of estimators proposed by Gart and Buck [4] to estimate the sensitivity and specificity of the IT are:

2 $$ {Sn}_{cor}^{GB}=\frac{S{p}_{RS}\times Prr\times S{n}_{IT}+\left(1-S{p}_{RS}\right)\left(1- Prr\right)\times S{p}_{IT}-\left(1-S{p}_{RS}\right)\left(S{p}_{RS}-\hat{P}J\right)}{\hat{P}J} $$

3 $$ S{p}_{cor}^{GB}=\frac{S{n}_{RS}\times \left(1- Prr\right)\times S{p}_{IT}+\left(1-S{n}_{RS}\right)\times Prr\times S{n}_{IT}-\left(1-S{n}_{RS}\right)\left(1-S{p}_{RS}+\hat{P}J\right)}{J\left(1-\hat{P}\right)} $$

#### Staquet et al. [5] correction method

Staquet et al. [5] proposed two pairs of estimators to estimate the sensitivity and specificity of the IT under two scenarios. The first pair of estimators (to estimate the sensitivity and specificity of the IT) is proposed for when the IT and RS are conditionally independent and the sensitivity and specificity of the RS are known. A second pair of estimators (to estimate the sensitivities of the IT and RS) is proposed when the specificities of the IT and RS are perfect (100%). In this paper, we focus on the first pair of estimators. This pair of estimators is employed to estimate the sensitivity and specificity of the IT given that the IT and the RS are conditionally independent and the sensitivity and specificity of the RS are known. These estimators are:

4 $$ S{n}_{cor}^{sq}=\frac{gS{p}_{RS}-b}{N\left(S{p}_{RS}-1\right)+e};\kern0.5em S{p}_{Cor}^{sq}=\frac{hS{n}_{RS}-c}{NS{n}_{RS}-e};\kern0.5em \hat{P}=\frac{N\left(S{p}_{RS}-1\right)+e}{N\left(S{n}_{RS}+S{p}_{RS}-1\right)}\kern1em $$

The Staquet et al. [5] correction method is equivalent to the Gart and Buck [4] correction method (see Additional file 1).

#### Brenner [6] correction method

Brenner [6] proposed two pairs of estimators to estimate the sensitivity and specificity of the IT. The first pair of estimators assumes that the IT and the RS are conditionally independent and the second pair of estimators assumes that the IT and RS are conditionally dependent (positively correlated) given the true disease status of the individuals. In both pairs of estimators, the sensitivity and specificity of RS are assumed known. However, in this paper, we focus on the first pair of estimators, where the IT and RS are assumed to be conditionally independent. The first pair of estimators is expressed as:

5 $$ S{n}_{cor}^{B1}=\frac{Prr\times S{n}_{RS}\times S{n}_{IT}+\left(1-\mathrm{Prr}\right)\left(1-{\mathrm{Sp}}_{\mathrm{RS}}\right)\left(1-S{p}_{IT}\right)}{Prr\times S{n}_{RS}+\left(1- Prr\right)\left(1-S{p}_{RS}\right)} $$

6 $$ S{p}_{cor}^{B1}=\frac{Prr\times \left(1-S{n}_{RS}\right)\left(1-S{n}_{IT}\right)+\left(1- Prr\right)\times S{p}_{RS}\times S{p}_{IT}}{Prr\times \left(1-S{n}_{RS}\right)+\left(1- Prr\right)\times S{p}_{RS}} $$

The two estimators (5) & (6) can be re-written as (7) & (8)  respectively (see Additional file 1)

7 $$ S{n}_{cor}^{B1}=\frac{aS{n}_{RS}+b\left(1-S{p}_{RS}\right)}{eS{n}_{RS}+f\left(1-S{p}_{RS}\right)} $$

8 $$ S{p}_{cor}^{B1}=\frac{c\left(1-S{n}_{RS}\right)+ dS{p}_{RS}}{e\left(1-S{n}_{RS}\right)+ fS{p}_{RS}} $$

### Simulation study

The correction methods were compared using simulation techniques and analysis of clinical datasets. Since the Staquet et al. [5] approach is equivalent to the Gart and Buck [4] correction method (see Additional file 1), only the Staquet et al. [5] approach was compared to the Brenner correction method. The simulation was conducted following the guidelines by Morris et al. [10] which include **P**lanning for the simulation, **C**oding and execution, **A**nalysis and **R**eporting the simulation study appropriately (PCAR), and using R-Studio statistical software [11]. In the simulation, the Staquet et al. [5] and Brenner [6] approaches were compared with the classical method [12]. The classical method assumes that the reference standard is a gold standard (Eq. (1)). The estimates obtained from the classical method will be called unadjusted estimates of sensitivity and specificity.

The performance measures in the simulation are the basic statistical properties used to ascertain a good estimator. These properties are unbiasedness, mean square error (MSE) and consistency. Further notes on these properties are presented in Additional file 1.

The fixed effects [1, 13] modelling approach was employed to simulate the different datasets using the multinomial distribution (“rmulti” function in R [14]). This approach models the pairwise conditional dependence (or correlation) between two tests among the diseased and non-diseased groups using covariance terms which are fixed across participants [15]. In the simulation process, the sensitivity and specificity of the IT and RS are known, and the prevalence of the target condition is known. The template showing how the cell probabilities were calculated using the prevalence, sensitivity and specificity of the IT and RS, and covariance terms is reported in Additional file 1 – Table S1.

The simulation study was carried out under two assumptions given that the RS is imperfect. Firstly, the IT and RS were assumed to be conditionally independent and secondly, the tests were assumed to be conditionally dependent. Theoretically, when the RS is error-free (perfect), the classical and correction methods estimate the sensitivity and specificity of the IT accurately. This is shown algebraically in Additional file 1.

Under the assumption that the RS and IT are conditionally independent, multiple (200) random samples of different sample sizes from 50 to 1000 were simulated using the multinomial distribution under three scenarios, which are:

- Scenario one: The RS is assumed to be better than the IT. This implies that the sensitivity and specificity of the RS are higher than the sensitivity and specificity of the IT.
- Scenario two: The IT is assumed to be better than the RS. This implies that the sensitivity and specificity of the IT are higher than the sensitivity and specificity of the RS.
- Scenario three: The sensitivity and specificity of RS and IT are assumed to be the same.

#### Scenario one

**Fig. 1:**
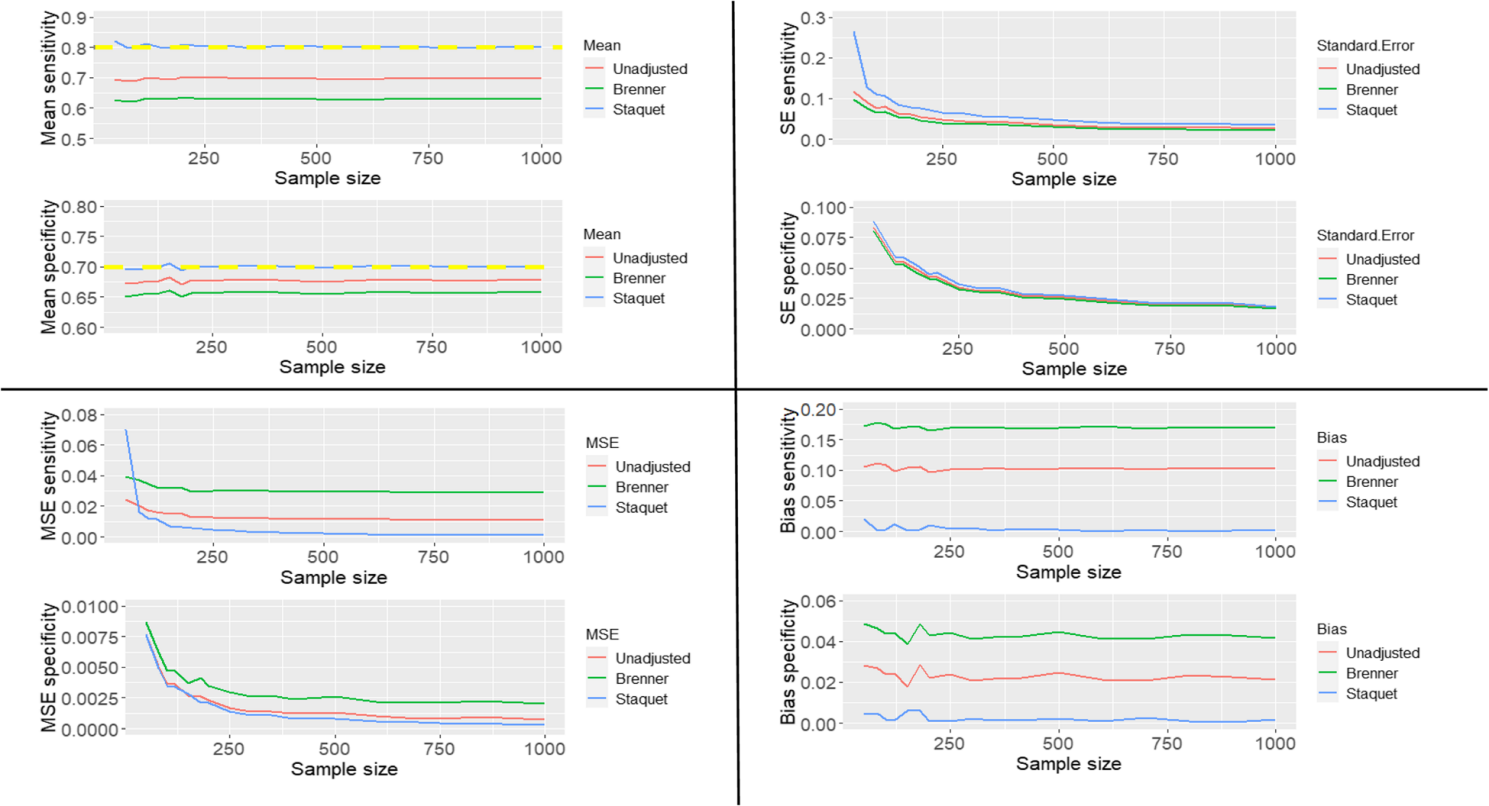
The mean, standard error, mean square error and bias of the unadjusted and corrected sensitivity and specificity of the index test when the reference standard is imperfect and better than the index test

The sensitivity and specificity of the RS are assumed to be 0.9 and the sensitivity and specificity of index test are 0.8 and 0.7 respectively. The prevalence is assumed to be 0.3. The unadjusted and corrected estimates are presented in Fig. 1.

#### Scenario two

**Fig. 2:**
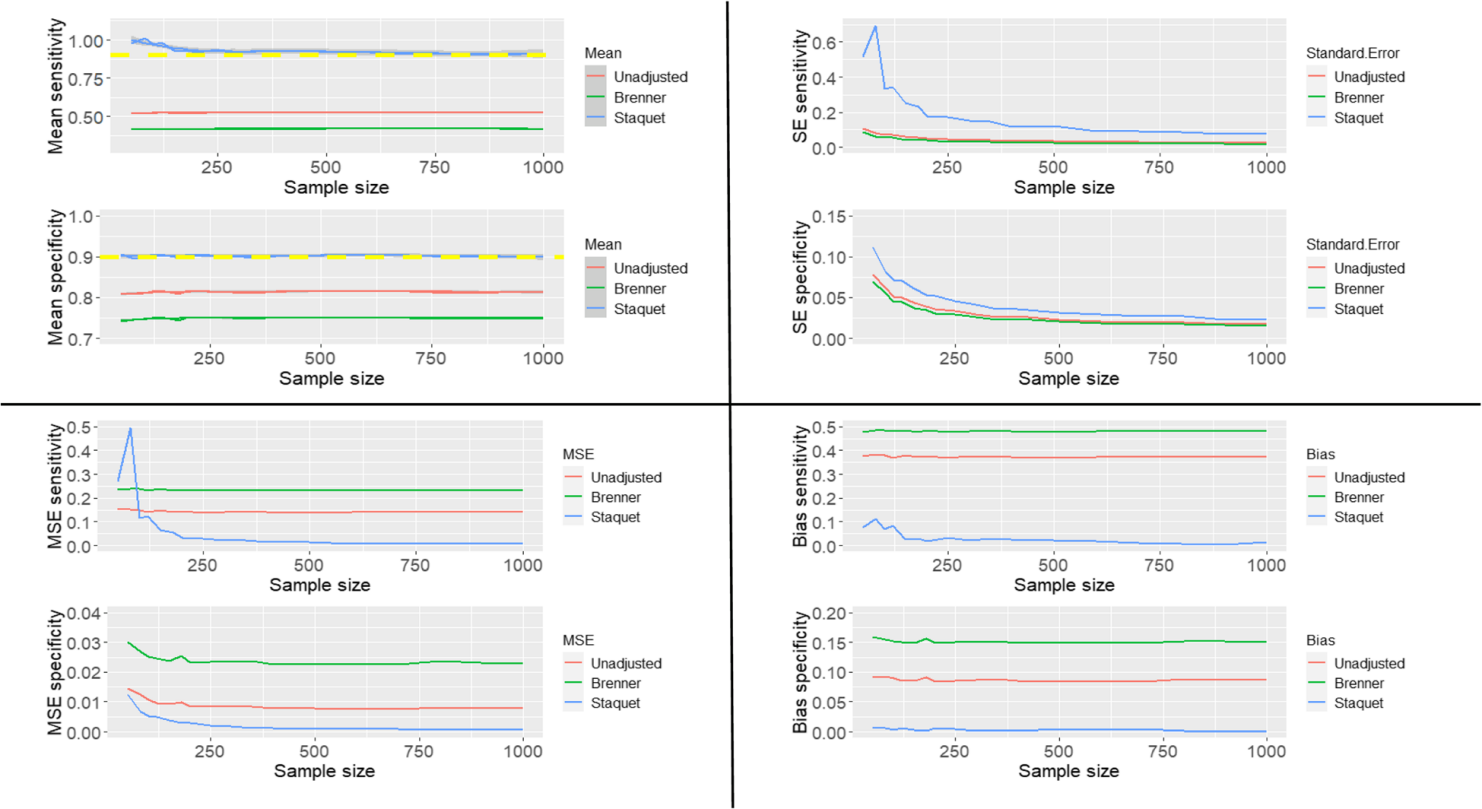
The mean, standard error, mean square error and bias of the unadjusted and corrected sensitivity and specificity of the index test when the reference standard is imperfect and worse than the index test

The sensitivity and specificity of the index test are 0.9 and the sensitivity and specificity of the reference standard are 0.8 and 0.7 respectively. The prevalence is 0.3. The unadjusted and corrected estimates are presented in Fig. 2.

#### Scenario three

**Fig. 3:**
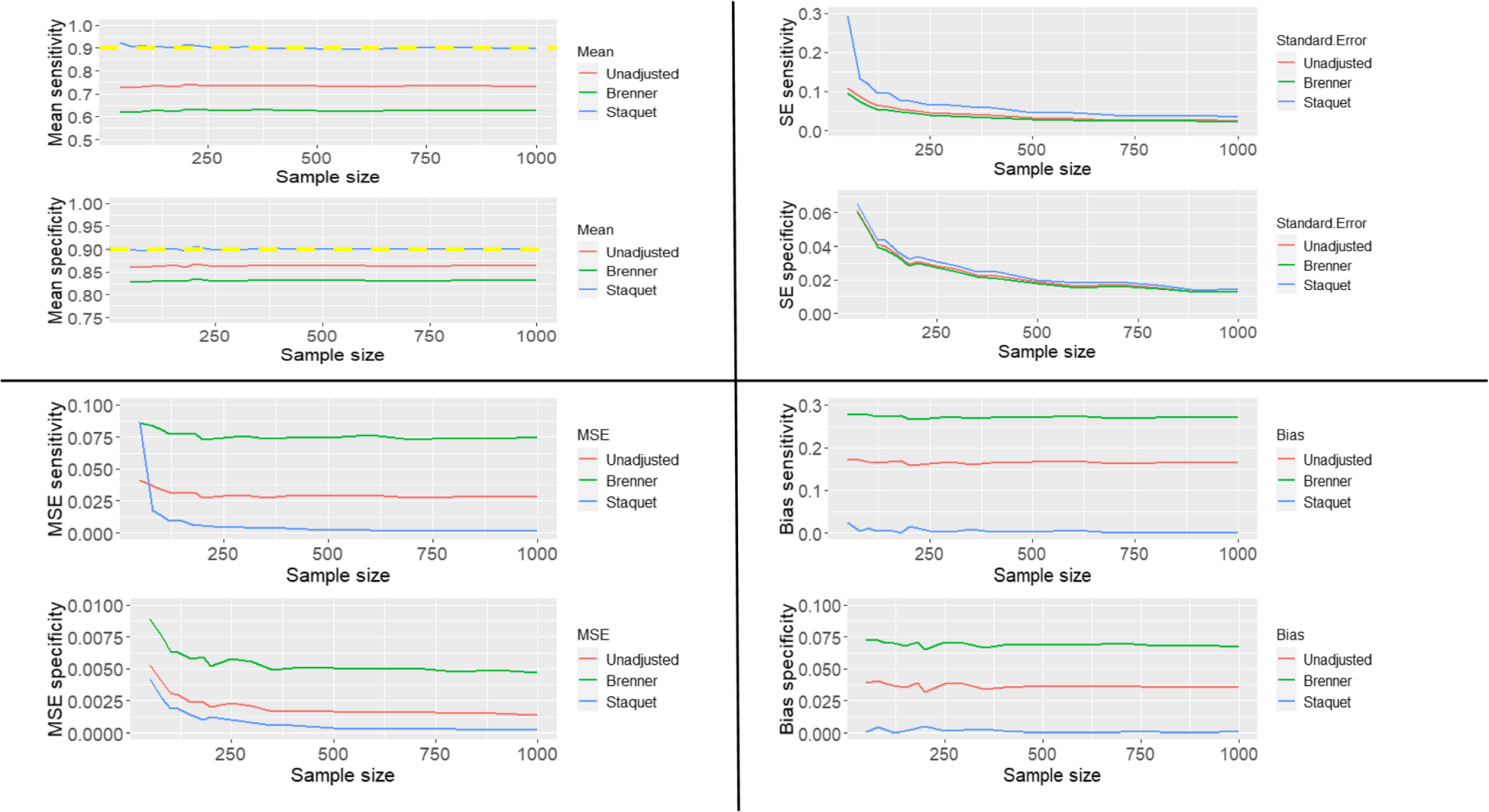
The mean, standard error, mean square error and bias of the unadjusted and corrected sensitivity and specificity of the index test when the reference standard is imperfect and has same diagnostic accuracy measures as the index test

The sensitivity and specificity of the index and reference tests are all 0.9, and the prevalence of the target condition is 0.3. The unadjusted and corrected estimates are presented in Fig. 3.

The yellow dashed lines on the plots of the mean sensitivities and mean specificities in Fig. 1 – Fig. 5 are the simulated true values of the sensitivity and specificity of the IT. The choice of parameters for the sensitivities and specificities of the IT and RS, and prevalence of the target condition were informed by clinical case studies identified from the review previously conducted by Chikere et al. [3].

Based on the three scenarios simulated, the estimates obtained from the Staquet et al. [5] method are accurate irrespective of which test is better or worse than the other. However, when the accuracy measures of the index test are better than the sensitivity and specificity of the reference standard (Fig. 2), a relatively large sample size (*n* > 200) would be recommended; as using small sample sizes produced mean sensitivities that were slightly above the simulated true value (0.9). Practically, information about the index test is usually unknown, so using relatively a large sample size in diagnostic accuracy study is typical. The unadjusted and Brenner corrected sensitivities are consistently lower than the simulated true value and the bias is consistently greater than 0.1. The unadjusted specificities are typically slightly below the simulated true values, and the bias is typically relatively small, below 0.05, except the bias from scenario two (Fig. 2), which is larger than 0.05 but below 0.1. The Brenner corrected specificities are consistently below the simulated true values and the bias is consistently above 0.1, except in scenario one (Fig. 1), in which is below 0.05. Further scenarios explored include cases where the sensitivity (or specificity) of the index test was better than the sensitivity (or specificity) of the RS. The results of these simulations are reported in Additional file 1. With the simulated scenarios explored, (given that the IT and RS are conditionally independent), the Staquet et al. [5] correction method outperforms the Brenner correction method. There could be other possible scenarios where the sensitivity (or specificity) of the IT and RS, and prevalence is not equivalent to the values explored in this paper. These scenarios can be explored using the R-Code written by the Authors. The R-Code employed to generate and analyse the simulated and clinical datasets is presented in the appendix (Additional file 2).

**Fig. 4:**
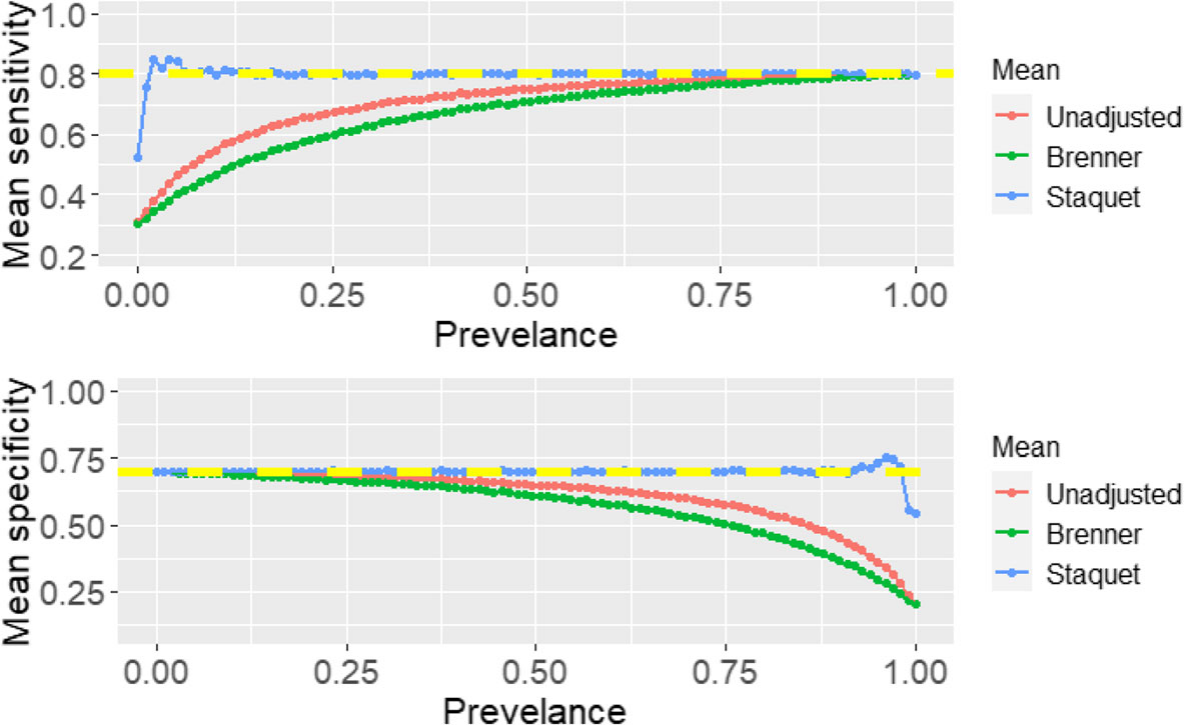
Unadjusted and corrected sensitivity and specificity of the index test under varying prevalence

In the three scenarios above, the simulation process only looked at a single prevalence (*p* = 0.3). Further analyses were carried out to explore the correction methods under varying prevalences ranging from 0 to 1 (in increments of 0.01) using scenario one, where the RS is better than the IT. The unadjusted and corrected sensitivity and specificity of the IT are presented in Fig. 4. Only scenario one was explored further because, in each scenario above, the Staquet et al. [5] correction method outperforms the Brenner correction and classical methods.

From Fig. 4 the unadjusted and Brenner corrected sensitivities tend towards the simulated true value as the prevalence tends to one. The Brenner corrected and unadjusted specificities tend to the simulated truth as the prevalence tends to zero. The Staquet et al. [5] sensitivity and specificity of the IT are approximately unbiased and equivalent to the simulated true value irrespective of the prevalence indicating a constant sensitivity and specificity across populations with different prevalences. However, when the prevalence is very low (< 0.1) or very high (> 0.9), there is the possibility of obtaining illogical estimated sensitivity or specificity via the Staquet et al. [5] approach. Illogical results imply that the estimated sensitivity or specificity is greater than one or less than zero. In the simulated datasets generated to produce Fig. 4, when the prevalence was 0.01 and 0.02 the estimated mean sensitivities were 5.38 × 10^12^ and 4.33 × 10^12^ respectively. These values are illogical and excluded from the plot. In addition, in the simulated datasets employed to plot Fig. 3, when the sample size is 50 and 80, the mean estimated sensitivities were − 2.04 × 10 [13] and − 1.94 × 10 [13], these values were also excluded from the plot. The simulated datasets are reproducible using the R-Code reported in Additional file 2. Further exploration of illogical estimates obtained via the Staquet et al. [5] correction method are discussed in Additional file 3.

**Fig. 5:**
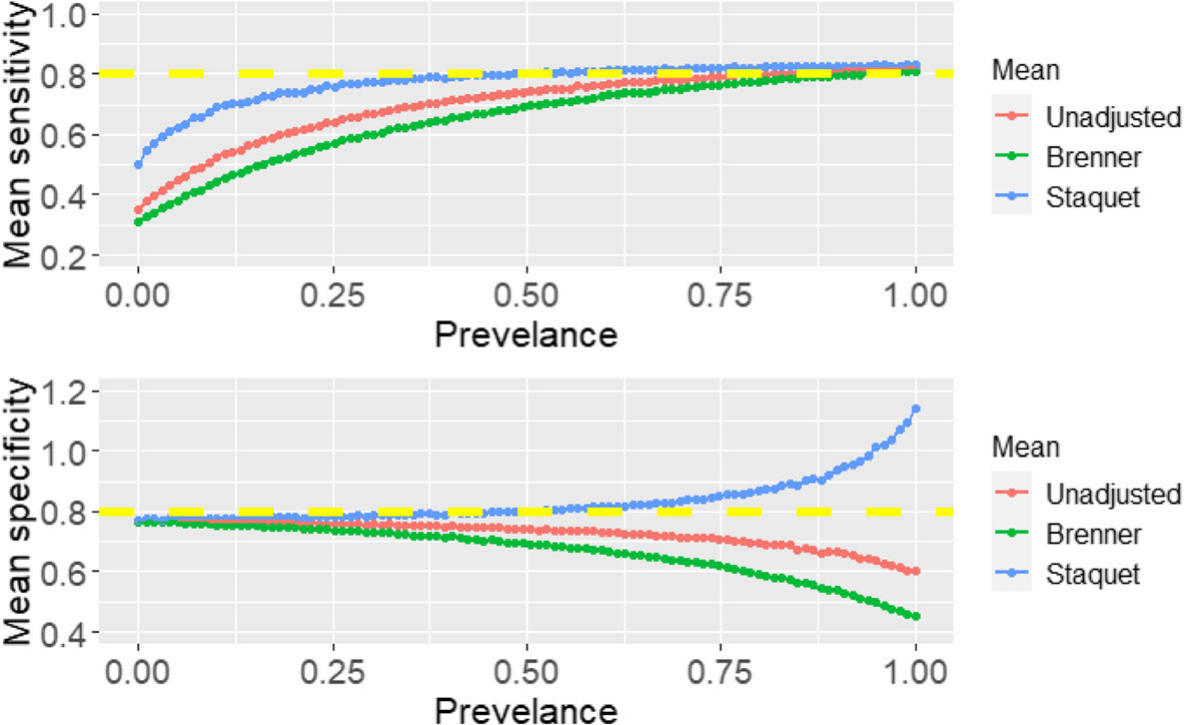
The mean unadjusted and corrected sensitivity and specificity of the index test when the reference standard is imperfect and the index test and reference standard are positively correlated

Under the assumption that the IT and RS are conditionally dependent, the covariance terms among the disease and non-disease groups are non-zero and they are varied to represent a selection of the diverse possible scenarios. A possible scenario is a case where the IT and the RS are positively correlated. In such case, the covariance terms among the diseased and non-diseased groups are positive. With the sensitivity and specificity of IT as 0.8 and the sensitivity and specificity of RS as 0.9, a scenario explored is where the covariance term among the diseased and non-diseased group is 0.05. The choice of covariance terms is constrained inequality constraint employed in generating the simulated datasets using the fixed effects modelling approach [1, 13]. The unadjusted and corrected estimates are presented in Fig. 5.

Further scenarios were explored and are reported in Additional file 4. Under the assumption of conditional dependence between IT and RS, all the correction methods performed poorly in the scenarios explored (see Additional file 4). This is expected, as the approaches were not developed to estimate the accuracy measures of the index test when the index test and the reference standard are conditionally dependent. However, when the covariance term between the disease groups is relatively small (close to zero), the Staquet et al. [5] correction method outperforms the Brenner correction method. In addition, when the IT and RS are conditionally dependent, the estimated sensitivity and specificity of the index test obtained via the Staquet et al. [5] correction method are not constant across different populations with varying prevalences, compared to the estimates obtained when the IT and RS are conditionally independent.

## Analysis of three clinical datasets

Three clinical datasets from two published articles (Mathews et al. [16] and Matos et al. [17]) were analysed to understand the impact of the choice of method in clinical decision making, and to support the findings from the simulation studies. The 95% confidence intervals of the estimates obtained were calculated using the Wilson score interval approach [18].

### Analysis of the clinical dataset from Mathews et al. [16] (case-study one)

The extracted clinical dataset from Mathews et al. [16] (Table *3*) aims to estimate the sensitivity and specificity of high resolution anoscopy (HRA) cytology in discriminating HIV patients into high grade squamous intraepithelial lesion (HSIL) and atypical squamous cells cannot rule out high grade (ASC-H) or not.

**Table 3:** Results of HRA cytology and punch biopsy in classifying patients into high grade and non-high grade squamous intraepithelial lesion

	Biopsy ***≥AIN***2	Biopsy ***<AIN***2	Total
Cytology HSIL or ASC-H	40	22	62
Cytology < HSIL	22	177	199
	62	199	261

*HSIL* High grade squamous intraepithelial lesion, *ASC-H* Atypical squamous cells cannot rule out high grade, *AIN* Anal intraepithelial neoplasia

The punch biopsy was employed as the RS but it is known to be imperfect. According to Mathews et al. [16], the sensitivity and specificity of punch biopsy were extracted from Byrom et al. [19] and are 0.74 and 0.91 respectively. The study employed the Staquet et al. [5] approach to correct for the sensitivity and specificity of HRA cytology given that the accuracy measures of punch biopsy are known and assuming the tests (IT and RS) are conditionally independent. The dataset (Table 3) was reanalysed using the Brenner correction method. The estimated prevalence is 0.27 and the corrected and unadjusted sensitivity and specificity estimates of HRA cytology are presented in Table 4.

**Table 4:** Unadjusted and corrected sensitivities and specificities of HRA cytology

Accuracy measures	Methods		
	Unadjusted (95% CI)	Brenner (95% CI)	Staquet et al (95% CI)
Sensitivity	0.65 (0.52, 0.75)	0.50(0.38, 0.62)	0.89 (0.79, 0.95)
Specificity	0.89 (0.84, 0.93)	0.85 (0.79, 0.89)	0.96 (0.92, 0.98)

*CI* Confidence interval

From Table 4, the estimated sensitivity and specificity of HRA cytology obtained via the Staquet et al. [5] approach are higher than the estimates obtained via the classical and Brenner correction methods. In addition, no illogical estimates were obtained via the Staquet et al. [5] approach. Furthermore, the confidence intervals from the Brenner and Staquet et al. [5] correction methods do not overlap. In this clinical application, correcting the sensitivity and specificity of HRA cytology using the Brenner approach would underestimate the sensitivity of HRA cytology; thus, discouraging its use to rule out the diagnosis of HSIL as the sensitivity would appear poor (0.5). However, correcting the diagnostic accuracy measures of HRA cytology using the Staquet et al. [5] approach encourages the use of HRA cytology in clinical practice to rule in the diagnosis of HSIL as it has a specificity that is close to one.

### Analysis of two clinical datasets from Matos et al. [17] dataset – (case-study two)

The extracted datasets from Matos et al. [17] reported in Table *5* and Table *6* are from Examiner 1 and the aim is to estimate the sensitivity and specificity of fluorescence – based devices (Fluorescence camera – FC and DIAGNOdent – a pen type laser fluorescence abbreviated as LFpen) used in detecting occlusal caries lesions in primary teeth. The study used the Brenner [6] correction method to estimate the sensitivities and specificities under the assumption that the sensitivity and specificity of the RS (visual inspection) are known and that the fluorescence devices are conditionally independent of the RS. The two different target conditions are non-cavitated caries lesions (NC) and dentine caries lesions (D3).

**Table 5:** Results of the visual inspection (reference standard) and fluorescence - based devices (LFpen and FC) in discriminating teeth with non-cavitated lesions

	Reference standard (NC – Examiner 1)			Reference standard (NC – Examiner 1)	
Index test	Positive	Negative	Index test	Positive	Negative
LFpen positive	241	6	FC positive	156	3
LFpen negative	110	26	FC positive	195	29
	351	32		351	32

**Table 6:** Results of the visual inspection (reference standard) and fluorescence - based devices (LFpen and FC) in discriminating teeth with Dentine lesions

	Reference standard (D3 – Examiner 1)			Reference standard (D3 - Examiner 1)	
Index test	Positive	Negative	Index test	Positive	Negative
LFpen positive	20	45	FC positive	21	38
LFpen negative	1	341	FC positive	0	348
	21	386		21	386

Table 5 reports the classification of the results from the index tests (FC and LFpen) and reference standard when the target condition is NC and Table 6 reports the classification of the results from the index tests (FC and LFpen) and reference standard when the target condition is D3.

Matos et al. [17] obtained the diagnostic accuracy of the reference standards from previous studies [20–24]. For the NC detection, the sensitivity and specificity of the RS were 0.796 and 0.799 respectively. For the D3, the sensitivity and specificity of the RS were 0.786 and 0.995 respectively. In addition, the teeth were assumed to be independent.

The unadjusted and corrected sensitivities and specificities of the LFpen and FC in discriminating between teeth with NC (Table 5) are presented in Table 7.

**Table 7:** Unadjusted and corrected sensitivities and specificities of LFpen and FC in detection of NC

	Methods		
Accuracy measures	Unadjusted (95% CI)	Brenner (95% CI)	Staquet et al (95% CI)
**Non-cavitated caries lesion (NC) – LFpen**			
Sensitivity	0.69 (0.64, 0.73)	0.68 (0.63, 0.73)	0.70 (0.65, 0.75)
Specificity	0.81 (0.65, 0.91)	0.44 (0.28, 0.61)	0.04 (0.01, 0.17)
**Non-cavitated caries lesion (NC) – FC**			
Sensitivity	0.44 (0.39, 0.50)	0.44 (0.39, 0.49)	0.45 (0.40, 0.50)
Specificity	0.91 (0.76, 0.97)	0.65 (0.48, 0.79)	0.36 (0.22, 0.53)

*CI* Confidence interval, *LFpen* Laser florescence pen, *FC* Fluorescence camera

The sample prevalence for NC (0.92) and the estimated prevalence via the Staquet et al. [5] approach is 1.2 (which is illogical). An illogical prevalence is explored in Additional file 3; it was observed that when the sensitivity of the reference standard is less than the sample prevalence, illogical prevalence is likely to be obtained. The estimated sensitivities for LFpen (≅ 0.7) and FC (0.44 or 0.45) are consistent across all methods (Table 7), and the confidence intervals from the corrected and unadjusted sensitivities overlap. The specificities of LFpen and FC differ across the methods, with the Staquet et al. [5] corrected specificities (LFpen is 0.04, and FC is 0.36) being the lowest of all. At so a high prevalence and in particular when the estimated prevalence is illogical (1.2), the estimated specificity via the Staquet et al. [5] should be treated with scepticism.

The second dataset from Matos et al. [17] (Table 6) was analysed; the estimated prevalence is 0.06 and the sample prevalence is 0.052. The unadjusted and corrected sensitivity and specificity of the LFpen and FC in discriminating between teeth D3 are presented in Table 8.

**Table 8:** Unadjusted and corrected sensitivities and specificities of LFpen and FC in detecting D3

	Methods		
Accuracy measures	Unadjusted (95% CI)	Brenner (95% CI)	Staquet et al (95% CI)
**Dentine caries lesion (D3) – LFpen**			
Sensitivity	0.95 (0.77, 0.99)	0.86 (0.66, 0.95)	1.04 (NaN)
Specificity	0.88 (0.85, 0.91)	0.87 (0.83, 0.90)	0.90 (0.87, 0.93)
**Dentine caries lesion (D3) – FC**			
Sensitivity	1.00 (0.85, 1.00)	0.91 (0.72, 0.98)	1.09 (NaN)
Specificity	0.90 (0.87, 0.93)	0.89 (0.86, 0.92)	0.92 (0.89, 0.94)

*CI* Confidence interval, *LFpen* Laser florescence pen, *FC* Fluorescence camera, *NaN* Not available or cannot be estimated

From Table 8, the estimated and sample prevalence of D3 are very low (< 0.1), hence the specificities of LFpen and FC (≅ 0.9) are consistent across all methods and are estimated accurately, and the confidence intervals from the corrected and unadjusted specificities overlap. However, the sensitivities differ, with Staquet et al. [5] providing illogical estimates (estimates greater than 1). Given the illogical results, the 95% confidence intervals cannot be estimated. Obtaining illogical estimates for the sensitivity of FC and LFpen via the Staquet et al. [5] approach are in line with the observations from the simulation study.

## Discussion

### Simulation study

Firstly, when the RS is perfect (*Sn*_*RS*_ = *Sp*_*RS*_ = 1), the estimates obtained from all the correction methods and the classical method are the same (this is expressed algebraically in Additional file 1). Secondly, when the RS is imperfect and the RS is conditionally independent of the IT, the Staquet et al. [5] correction method outperforms the Brenner correction method irrespective of which test is better. In addition, the estimates obtained via the Staquet et al. [5] correction method uphold the assumption of constant sensitivity and specificity across populations with different prevalence. This implies that the disease prevalence in the population does not affect the estimates obtained via the Staquet et al. [5] method unlike the classical and the Brenner correction methods. At a low prevalence, the estimated sensitivity from the classical and Brenner methods are often underestimated and at high prevalence the estimated specificity from the classical and Brenner correction method are typically underestimated. Thus, when there is a high prevalence, the sensitivity is more likely to be accurately estimated by all methods and at low prevalence, the specificity is likely to be accurately estimated by all methods. This is consistent with findings reported by other researchers that in a high prevalence population the sensitivity of the index test is often likely to be estimated accurately and in a low prevalence population the specificity is likely to be estimated accurately [1, 25]. Furthermore, not correcting for the imperfection of the IT using the classical method yield estimates that are closer to the simulated truth than correcting for the imperfection of the IT using the Brenner correction method. Thus, the classical method performs better than the Brenner correction method. Furthermore, when the IT and RS are conditionally dependent, both the Staquet et al. [5] and Brenner correction methods perform poorly.

### Analysis of the clinical datasets

The clinical datasets explored had varying prevalences, which aid in the exploration of the methods in clinical applications. The Mathews et al. [16] dataset had a sample prevalence of 0.23, and the two datasets from Matos et al. [17] had very low (0.052) and very high (approximately 0.92) sample prevalences. Using clinical datasets with varying prevalences supported the findings from the simulation study.

The analysis of the clinical datasets alongside the simulation study have shown that the prevalence of the target condition can cause illogical estimates for the sensitivity and specificity of the IT via the Staquet et al. [5] approach. However, there could be alternative rationale that could be considered if this occurs, for example that the two tests (IT and RS) are mathematically conditionally dependent, even though the IT and RS do not use the same biological component. However, we cannot conclude that obtaining an illogical estimate via the Staquet et al. [5] approach is a sufficient condition to establish that the IT and RS are conditionally dependent given the true disease status. The information in Additional file 3 – Table S2 shows that illogical estimates can be obtained via the Staquet et al. [5] approach when the tests are conditionally independent.

In scenarios where the estimates obtained via the Staquet et al. [5] approach are illogical, the traditional latent class model [7, 26–29] could be employed and the known sensitivity and specificity of the RS would be used as the priors (deterministic or probabilistic) in the model to estimate the accuracy measures of the IT. The latent class model has the advantage over the Staquet et al. [5] approach in that it does not produce illogical estimates. In addition, if the tests are conditionally dependent a Bayesian latent class model [15] could be considered.

One of the limitations of this study is that the traditional latent class model is not explored, as it is a probabilistic modelling approach and is not the focus of this work. In addition, the coverage probability of confidence intervals was not explored because it is a property of the procedure producing the confidence interval and not the estimators themselves, and as such is outside the scope of this study. Furthermore, there could be other scenarios (possible combinations of sensitivities and specificities of RS and IT, and prevalence) not explored in this paper; however, the R-Code (Additional file 2) written by the Authors would aid researchers who wish to explore more. A further area of research is to explore the Staquet et al. [5] approach to understand if there is a way it can indicate conditional dependence between two tests. Furthermore, as observed from the analysis of the clinical datasets (Table 5, Table 7 and Additional file 3), an illogical value was obtained for the estimated prevalence, which could have impacted the estimated specificities of the index tests. Thus, the Staquet et al. [5] approach could be further explored to ascertain other conditions that can make the Staquet et al. [5] approach produce illogical estimates, as well as possible implications where multiple conditions are satisfied simultaneously.

## Conclusions

From the simulation study (using the scenarios explored in this paper) and the analysis of the clinical datasets, the Staquet et al. [5] correction method outperforms the Brenner correction method. However, when the prevalence of the target condition is very high (> 0.9) or low (< 0.1), or the tests employed in the diagnostic accuracy study are correlated (conditionally dependent) other statistical methods should be considered such as the latent class model (frequentist or Bayesian) to avoid obtaining illogical or inaccurate estimates. Furthermore, using poor estimates of the accuracy measures for the reference standard would affect the estimated corrected sensitivity and specificity of the index test.

## Supplementary Information

**Additional file 1.**

**Additional file 2.**

**Additional file 3.**

**Additional file 4.**

## Data Availability

Datasets generated and analysed are included in this published article [and R-Code in Additional file 2].
