# Supplementary material for: Comparative diagnostic accuracy studies with an imperfect reference standard – a comparison of correction methods
Source: BMC Med Res Methodol. 2021 Apr 12;21:67. doi: 10.1186/s12874-021-01255-4 (PMC8040223; doi:10.1186/s12874-021-01255-4)
Supplement: Supplementary file 1 — Additional file 1. [file 12874_2021_1255_MOESM1_ESM.pdf]

Comparative diagnostic accuracy studies with an imperfect reference standard – A comparison of correction methods

Chinyereugo M. Umemneku Chikere[1*], Kevin J. Wilson[2], A. Joy Allen[3], Luke Vale[1]

[1] Population Health Science Institute, Faculty of Medical Sciences, Newcastle University

[2] School of Mathematics, Statistics and Physics, Newcastle University

[3] National Institute for Health Research, Newcastle In Vitro Diagnostics Co-operative, Newcastle University

* Corresponding author

Email: cmuc1@leicster.ac.uk (CMUC)

1.1. Simplification of the Gart and Buck estimators to obtain the Staquet et al estimators

$$Sn_{cor}^{GB} = \frac{Sp_{RS} \times Prr \times Sn_{IT} + (1 - Sp_{RS})(1 - Prr) \times Sp_{IT} - (1 - Sp_{RS})(Sp_{RS} - \hat{P}J)}{\hat{P}J}$$

$$= \frac{Sp_{RS} \times \frac{e}{N} \times \frac{a}{e} + (1 - Sp_{RS})\left(\frac{f}{N} \times \frac{d}{f}\right) - (1 - Sp_{RS})(Sp_{RS} - Prr - Sp_{RS} + 1)}{J(\hat{P})}$$

$$= \frac{\frac{a}{N}(Sp_{RS}) + \frac{d}{N} - \frac{d}{N}(Sp_{RS}) - (1 - Sp_{RS})(1 - Prr)}{J\hat{P}}$$

$$= \frac{\frac{a}{N}(Sp_{RS}) + \frac{d}{N} - \frac{d}{N}(Sp_{RS}) - \frac{f}{N}(1 - Sp_{RS})}{J\hat{P}}$$

$$= \frac{\frac{a}{N}(Sp_{RS}) + \frac{d}{N} - \frac{d}{N}(Sp_{RS}) - \frac{f}{N} + \frac{f}{N}(Sp_{RS})}{J\hat{P}}$$

$$= \frac{\frac{a}{N}(Sp_{RS}) - \frac{d}{N}(Sp_{RS}) + \frac{f}{N}(Sp_{RS}) - \frac{f}{N} + \frac{d}{N}}{J\hat{P}}$$

$$= \frac{\frac{a - d + f}{N}(Sp_{RS}) + \frac{d}{N} - \frac{f}{N}}{J\hat{P}}$$

$$= \frac{\frac{a + b}{N}(Sp_{RS}) - \frac{b}{N}}{J\hat{P}}$$

$$= \frac{g(Sp_{RS}) - b}{N(Prr + Sp_{RS} - 1)}$$

$$= \frac{g(Sp_{RS}) - b}{N(Prr) + N(Sp_{RS} - 1)}$$

$$Sn_{cor}^{sq} = \frac{g(Sp_{RS}) - b}{N(Sp_{RS} - 1) + e}$$

$$Sp_{cor}^{GB} = \frac{Sn_{RS} \times (1 - Prr) \times Sp_{IT} + (1 - Sn_{RS})Prr \times Sn_{IT} - (1 - Sn_{RS})\left(1 - Sp_{RS} + \hat{P}J\right)}{J\left(1 - \hat{P}\right)}$$

$$= \frac{Sn_{RS} \times \frac{f}{N} \times \frac{d}{f} + (1 - Sn_{RS}) \times \frac{e}{N} \times \frac{a}{e} - (1 - Sn_{RS})(1 - Sp_{RS} + Sp_{RS} + Prr - 1)}{J\left(1 - \hat{P}\right)}$$

$$= \frac{\frac{d}{N}(Sn_{RS}) + \frac{a}{N}(1 - Sn_{RS}) - (1 - Sn_{RS})(Prr)}{J\left(1 - \hat{P}\right)}$$

$$= \frac{\frac{d}{N}(Sn_{RS}) + \frac{a}{N} - \frac{a}{N}(Sn_{RS}) - \frac{e}{N}(1 - Sn_{RS})}{J\left(1 - \frac{Prr + Sp_{RS} - 1}{J}\right)}$$

$$= \frac{\frac{d}{N}(Sn_{RS}) + \frac{a}{N} - \frac{a}{N}(Sn_{RS}) - \frac{e}{N} + \frac{e}{N}(Sn_{RS})}{\frac{J(J - Prr - Sp_{RS} + 1)}{J}}$$

$$= \frac{\frac{d - a + e}{N}(Sn_{RS}) + \frac{a}{N} - \frac{e}{N}}{J\left(\frac{Sp_{RS} + Sn_{RS} - 1 - Prr - Sp_{RS} + 1}{J}\right)}$$

$$= \frac{\frac{h}{N}(Sn_{RS}) - \frac{c}{N}}{Sn_{RS} - Prr}$$

$$= \frac{h(Sn_{RS}) - c}{N(Sn_{RS} - Prr)}$$

$$Sp_{Cor}^{sq} = \frac{h(Sn_{RS}) - c}{NSn_{RS} - e}$$

1.2. Algebraic expression to show that estimates obtained from the classical and correction methods are the same when the RS is perfect

In this section, the different estimators are explored to understand how they are all the same when the reference standard is perfect.

Mathematically, the first pair of Brenner estimators can be reduced to:

$$Sn_{cor}^{B1} = \frac{Prr \times Sn_{RS} \times Sn_T + (1 - \mathrm{Prr})(1 - \mathrm{Sp_{RS}})(1 - Sp_{IT})}{Prr * Sn_{RS} + (1 - Prr)(1 - Sp_{RS})}$$

$$= \frac{\left(\frac{e}{N} \times Sn_{RS} \times \frac{a}{e}\right) + \left(\frac{f}{N} \times (1 - Sp_{RS}) \times \frac{b}{f}\right)}{\frac{eSn_{RS}}{N} + \frac{f(1 - Sp_{RS})}{N}}$$

$$= \frac{\frac{1}{N}(aSn_{RS}) + \frac{1}{N}\left(b(1 - Sp_{RS})\right)}{\frac{1}{N}\left(eSn_{RS} + f(1 - Sp_{RS})\right)}$$

$$\boldsymbol{Sn_{cor}^{B1}} = \frac{\boldsymbol{aSn_{RS} + b(1 - Sp_{RS})}}{\boldsymbol{eSn_{RS} + f(1 - Sp_{RS})}}$$

$$Sp_{cor}^{B1} = \frac{Prr * (1 - Sn_{RS})(1 - Sn_T) + (1 - Prr)Sp_{RS} \times Sp_{IT}}{Prr(1 - Sn_{RS}) + (1 - Prr)Sp_{RS}}$$

$$= \frac{\left(\frac{e}{N}(1 - Sn_{RS}) \times \frac{c}{e}\right) + \left(\frac{f}{N} \times Sp_{RS} \times \frac{d}{f}\right)}{\frac{e(1 - Sn_{RS})}{N} + \frac{fSp_{RS}}{N}}$$

$$= \frac{\frac{1}{N}(c(1 - Sn_{RS})) + \frac{1}{N}(dSp_{RS})}{\frac{1}{N}(e(1 - Sn_{RS}) + fSp_{RS})}$$

$$\boldsymbol{Sp_{cor}^{B1}} = \frac{\boldsymbol{c(1 - Sn_{RS}) + dSp_{RS}}}{\boldsymbol{e(1 - Sn_{RS}) + fSp_{RS}}}$$

If the reference standard is perfect ($Sn_R = Sp_R = 1$) the Staquet et al and Brenner corrected estimators for sensitivity and specificity reduces to the classical estimator for sensitivity (Sn_T) and specificity (Sp_T).

Staquet et al estimators:

$$Sn_{cor}^{sq} = \frac{gSp_{RS} - b}{N\left(Sp_{RS} - 1\right) + e} = \frac{g - b}{e} = \frac{a + b - b}{e} = \frac{a}{e} = Sn_{IT}$$

$$Sp_{cor}^{sq} = \frac{hSn_{RS} - c}{NSn_{RS} - e} = \frac{h - c}{N - e} = \frac{c + d - c}{e + f \ - e} = \frac{d}{f} = Sp_{IT}$$

Brenner estimators are:

$$Sn_{cor}^{B1} = \frac{aSn_{RS} + b(1 - Sp_{RS})}{eSn_{RS} + f(1 - Sp_{RS})} = \frac{a}{e} = Sn_{IT}$$

$$Sp_{cor}^{B1} = \frac{c(1 - Sn_{RS}) + dSp_{RS}}{e(1 - Sn_{RS}) + fSp_{RS}} = \frac{d}{f} = Sp_{IT}$$

Where a, b, c, d, e, f, g, h and N are described on Table 2 (in the main text).

Therefore, when the reference standard is perfect:

$$Sn_{cor}^{sq} = Sn_{cor}^{B1} = Sn_{IT} \quad and \quad Sp_{cor}^{sq} = Sp_{cor}^{B1} = Sp_{IT}$$

1.3. Definition of some statistical properties – Bias, MSE, Consistency and the Wilson score interval

Bias: A good estimator is unbiased if the difference between the true value of the parameter (θ) and the expected value of the estimator ($E(\hat{\theta})$) is equal to zero[1, 2]; that is:

$$\theta - E(\hat{\theta}) = 0$$

MSE: A good estimator is expected to have a zero MSE. MSE is the average (mean) of the squared difference between the estimator and the true parameter[2]; that is:

$$E\left[(\theta - \hat{\theta})^2\right] = \frac{1}{n}\sum_{i=1}^{n}(\theta_i - \hat{\theta}_i)^2$$

Consistent: A good estimator is also expected to be consistent. An estimator is consistent if as the sample size increase ($n \rightarrow \infty$), the mean values of the estimator approaches the true values[2, 3]. That is:

$$E(\hat{\theta}) \rightarrow \theta \quad as \ n \rightarrow \infty; \quad n = sample \ size$$

In addition, it is expected that the variance of the estimator will decrease as the sample size increase. The empirical standard error of the simulation is calculated as[4]:

$$\sqrt{\frac{1}{n-1}\sum_{i=1}^{n}(\hat{\theta} - \bar{\theta})^2}$$

Calculation of the standard deviation of sensitivity and specificity of an index test and the confidence interval: The Wilson score interval[5, 6] formula was used to calculate the 95% confidence interval of the estimated sensitivity and specificity. Let $\hat{\theta}$ denote the estimated sensitivity or specificity of a test. The 95% confidence interval is calculated as:

$$(LL_{\hat{\theta}}, UL_{\hat{\theta}}) = \frac{1}{1 + \frac{z^2}{n_*}}\left(\hat{\theta} + \frac{z^2}{2n_*}\right) \pm \frac{z}{1 + \frac{z^2}{n_*}}\sqrt{\frac{\hat{\theta}(1-\hat{\theta})}{n_*} + \frac{z^2}{4n_*^2}}$$

$\boldsymbol{n}_*$ is not the total number of participants in the study. It is the total number of participants with positive (negative) test results on the reference standard when calculating the confidence interval of the estimated sensitivity (specificity).

Table S1: A 2 x 2 cell probabilities table classified by reference standard and index test result

	Reference standard	
Index test	Positive (+)	Negative (-)
Positive (+)	$p(Sn_{RS} \times Sn_{IT} + \varphi_{11\|1}) + (1-p)\left((1-Sp_{RS})(1-Sp_{IT}) + \varphi_{11\|1}\right)$	$p((1-Sn_{RS}) \times Sn_{IT} + \varphi_{01\|1}) + (1-p)(Sp_{RS}(1-Sp_{IT}) + \varphi_{01\|1})$
Negative (-)	$p(Sn_{RS}(1-Sn_{IT}) + \varphi_{10\|1}) + (1-p)(Sp_{IT}(1-Sp_{RS}) + \varphi_{10\|1})$	$p((1-Sn_{RS})(1-Sn_{IT}) + \varphi_{00\|1}) + (1-p)(Sp_{RS} \times Sp_{IT} + \varphi_{00\|1})$
P is prevalence, Sn_{IT} is the sensitivity of the index test; Sp_{IT} is the specificity of the index test; Sn_{RS} is the sensitivity of the reference standard, Sn_{RS} is specificity of the reference standard; $\varphi_{11\|1}, \varphi_{10\|1}, \varphi_{01\|1}. \varphi_{00\|1}$ are the covariance terms among the diseased group		

1.4. More simulated scenarios under the assumption that the IT and RS are conditionally independent.

In this section, other scenarios were considered to evaluate the Staquet et al[7] and Brenner[8] correction methods given that the RS is imperfect and the IT and RS are conditionally independent. The different scenarios are grouped into four groups. The first group used a prevalence fixed at 0.3 and fixed values for the sensitivities and specificities of RS and IT. The second group used fixed values for the sensitivities and specificities for IT and RS; however, the prevalence varied from 0 to 1 (in increments of 0.01). The third group used a prevalence that is fixed at 0.3; however, the sensitivity (or specificity) of RS or IT was varied. The fourth used a prevalence that is fixed at 0.7; however, the sensitivity (or specificity) of RS or IT was varied. For all scenarios explored, multiple (200) sample sizes of 1000 participants were simulated. The R-code is in Additional File 2. The yellow dashed line in all the figures are the simulated true values.

The first group of scenarios explored are:

a) **Scenario one**: The sensitivity of the IT (0.9) is better than the sensitivity of the RS (0.7) and their specificity are the same (0.9).
b) **Scenario two**: The specificity of the IT (0.9) is better than the specificity of the RS (0.7) and their sensitivity is the same (0.9).
c) **Scenario three**: The sensitivity of the RS (0.9) is better than the sensitivity of the IT (0.7) and their specificity is the same (0.9).
d) **Scenario three**: The specificity of RS the (0.9) is better than the specificity of the IT (0.7) and their sensitivity is the same (0.9).

The simulated true value for the prevalence is fixed at 0.3. The mean corrected and unadjusted sensitivity and specificity of the index test is reported in Figure 1 as (a), (b), (c) and (d) respectively. From Figure 1, the estimates obtained from the Staquet et al approach is equivalent to the simulated true values.

Figure 1: The unadjusted and corrected mean sensitivity and mean specificity of the index test when the reference standard is imperfect and the prevalence is fixed at 0.3.

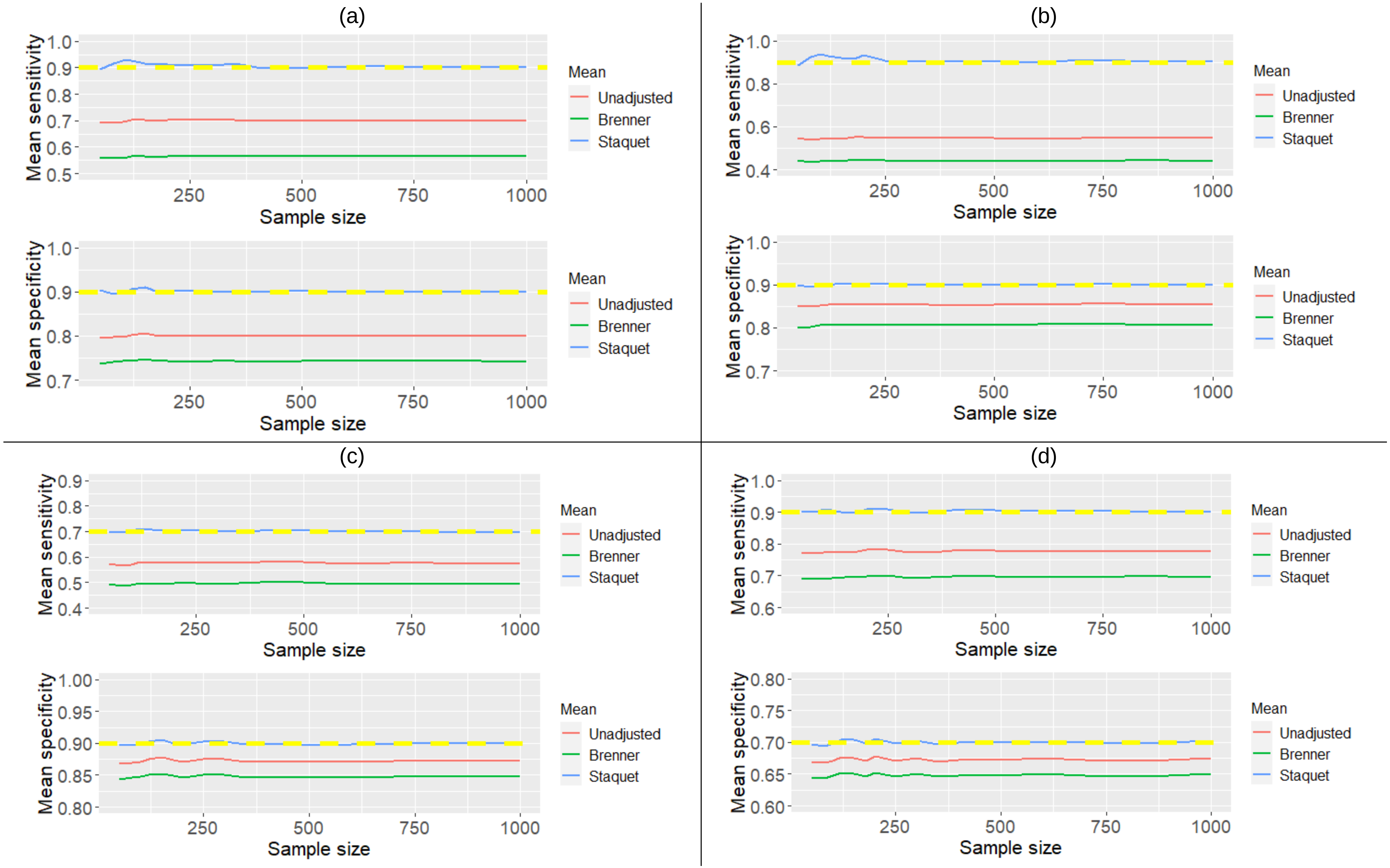

The second group of scenarios explored are:

a) **Scenario one**: The sensitivity of the IT (0.9) is better than the sensitivity of the RS (0.7) and their specificity are the same (0.9).

b) **Scenario two**: The specificity of the IT (0.9) is better than the specificity of the RS (0.7) and their sensitivity is the same (0.9).

c) **Scenario three**: The sensitivity of the RS (0.9) is better than the sensitivity of the IT (0.7) and their specificity is the same (0.9).

d) **Scenario three**: The specificity of RS the (0.9) is better than the specificity of the IT (0.7) and their sensitivity is the same (0.9).

The **prevalence is varied from 0 to 1 (in increments of 0.01**), the mean sensitivity and specificity of the IT in these four scenarios are reported in Figure 2 as (a), (b), (c), (d) respectively. From Figure 2, the estimates obtained from the Staquet et al correction method is equivalent to the simulated true values except at extreme side of the prevalence and the estimates are constant across the different prevalences.

Figure 2: The unadjusted and corrected mean sensitivity and mean specificity of the index test when the reference standard is imperfect, and the prevalences varies from 0 to 1.

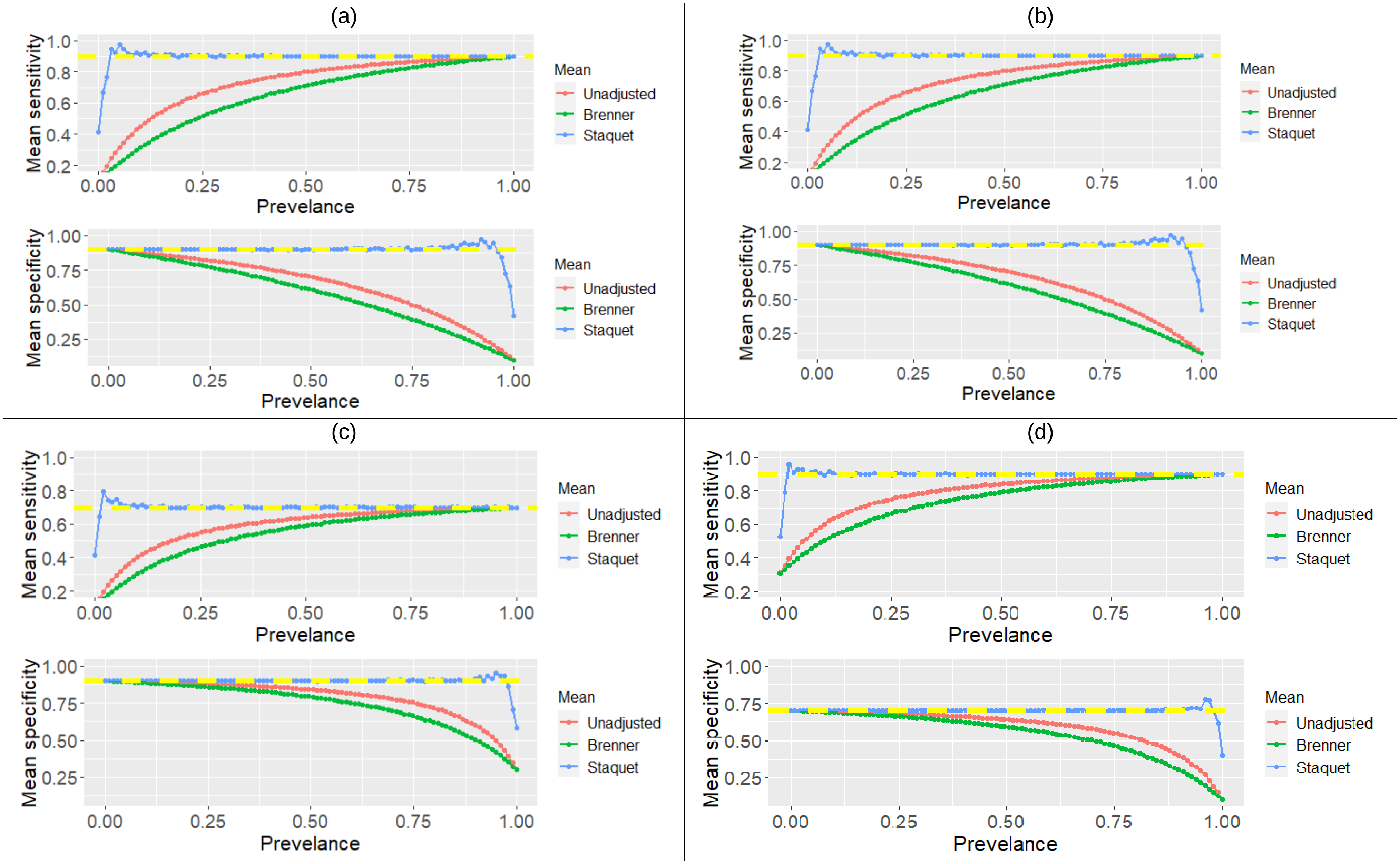

The third group of scenarios explored are:

a) **Scenario one**: The sensitivity of the RS was varied from 0 to 1 as the specificity of RS and IT, and the sensitivity of IT was fixed at 0.9, 0.8 and 0.8 respectively.

b) **Scenario two**: The specificity of the RS was varied from 0 to 1 as the sensitivity of RS and IT, and the specificity of IT was fixed at 0.9, 0.8 and 0.8 respectively.

c) **Scenario three**: The sensitivity of the IT was varied from 0 to 1 as the specificity of RS and IT, and the sensitivity of RS was fixed at 0.9, 0.8 and 0.9 respectively.

d) **Scenario three**: The specificity of the IT was varied from 0 to 1 as the sensitivity of RS and IT, and the specificity of RS was fixed at 0.9, 0.8 and 0.9 respectively.

The simulated true value for the **prevalence is fixed at 0.3**, the mean sensitivity and mean specificity of the IT in the four scenarios are reported in Figure 3 as (a), (b), (c), and (d) respectively. From Figure 3, the estimates obtained from the Staquet et al approach is equivalent to the simulated true values of the index test. However, when the sensitivity (or specificity) of the RS is very poor (< 0.3), the estimates obtained via the Staquet et al correction method could be inaccurate. Conventionally, the reference standard in clinical case studies do not have very poor accuracy measures. The sensitivity and specificity of a reference are often above 0.5.

Figure 3: The unadjusted and corrected mean sensitivity and mean specificity of the index test when the sensitivity (or specificity) of the reference standard or index test is varied and the prevalence is fixed at 0.3.

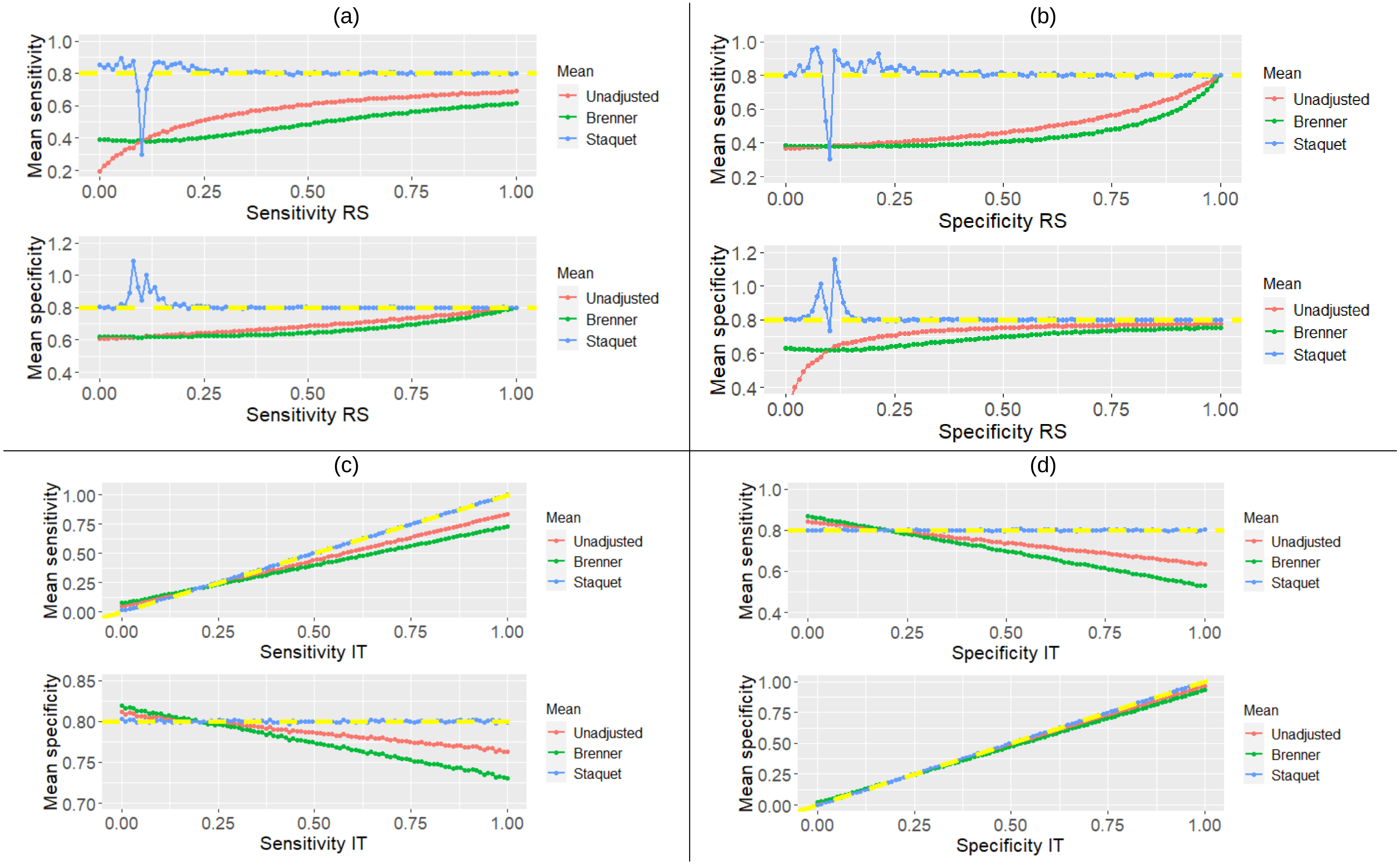

The fourth group of scenarios explored are:

a) **Scenario one**: The sensitivity of the RS was varied from 0 to 1 as the specificity of RS and IT, and the sensitivity of IT was fixed at 0.9, 0.8 and 0.8 respectively.

b) **Scenario two**: The specificity of the RS was varied from 0 to 1 as the sensitivity of RS and IT, and the specificity of IT was fixed at 0.9, 0.8 and 0.8 respectively.

c) **Scenario three**: The sensitivity of the IT was varied from 0 to 1 as the specificity of RS and IT, and the sensitivity of RS was fixed at 0.9, 0.8 and 0.9 respectively.

d) **Scenario three**: The specificity of the IT was varied from 0 to 1 as the sensitivity of RS and IT, and the specificity of RS was fixed at 0.9, 0.8 and 0.9 respectively.

The simulated true value for the **prevalence is fixed at 0.7**, the mean sensitivity and mean specificity of the IT in the four scenarios are reported in Figure 4 as (a), (b), (c), and (d) respectively. From Figure 4, the estimates obtained from the Staquet et al approach is equivalent to the simulated true values of the index test. However, when the sensitivity (or specificity) of the RS is very poor (< 0.3), the estimates obtained via the Staquet et al correction method could be inaccurate. Conventionally, the reference standard in clinical case studies do not have very poor accuracy measures. The sensitivity and specificity of a reference standard are often above 0.5.

<u>Figure 4:</u> The unadjusted and corrected mean sensitivity and mean specificity of the index test when the sensitivity (or specificity) of the reference standard or Index test is varied and the prevalence is fixed at 0.7.

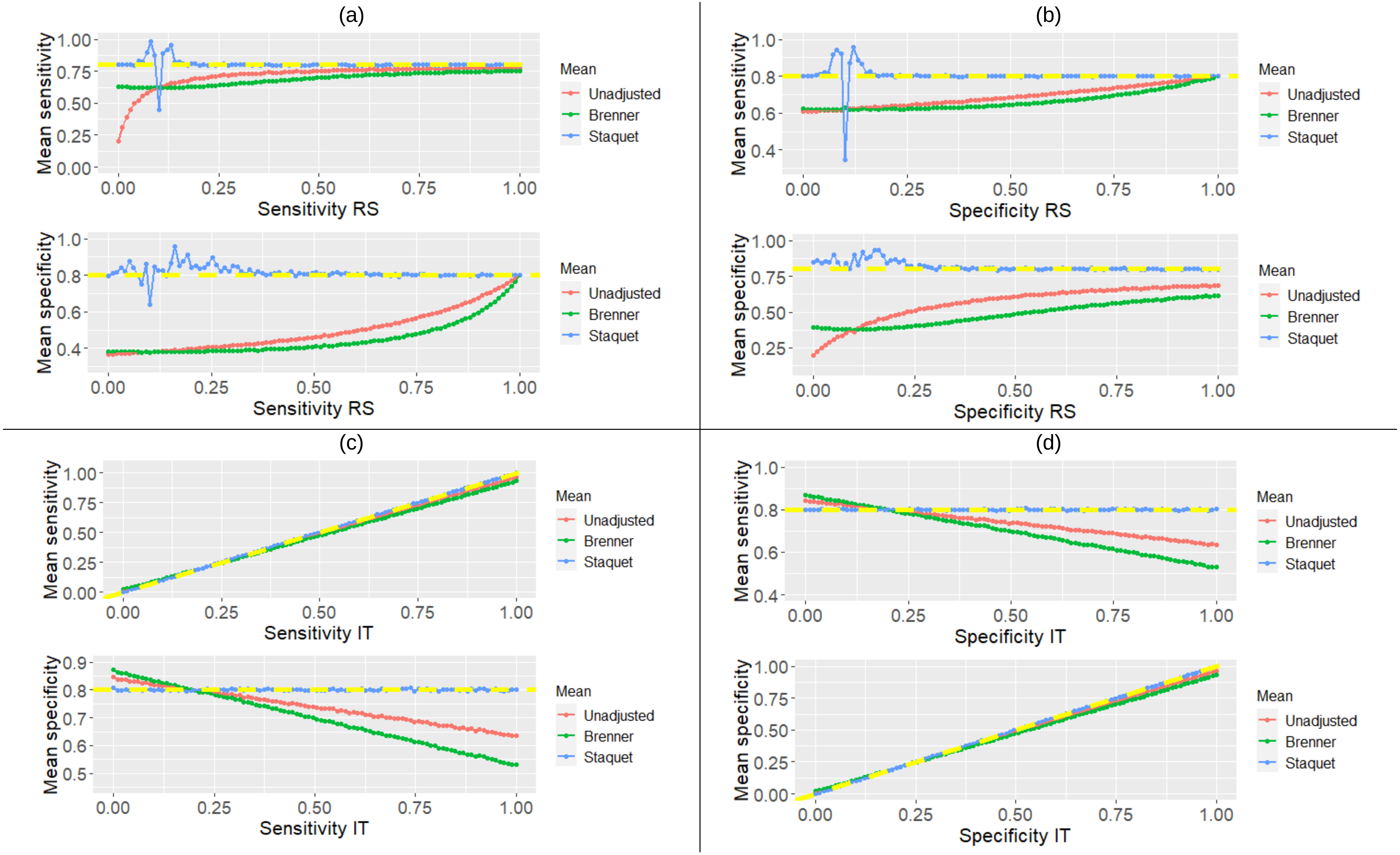

Reference

1. Voinov VGe and Nikulin MS. *Unbiased Estimators and Their Applications: Volume 1: Univariate Case*. Springer Science & Business Media, 2012.
2. Lehmann EL and Casella G. *Theory of point estimation*. Springer Science & Business Media, 2006.
3. Newey W. Chapter 36: Large sample estimation and hypothesis testing.(RF Engle and DL McFadden, eds.). Handbook of Econometrics 4 2111–2245. Elsevier, Edition, 1994.
4. Morris TP, White IR and Crowther MJ. Using simulation studies to evaluate statistical methods. *Statistics in medicine* 2019.
5. Wilson EB. Probable inference, the law of succession, and statistical inference. *Journal of the American Statistical Association* 1927; 22: 209-212.
6. Newcombe RG. Interval estimation for the difference between independent proportions: comparison of eleven methods. *Statistics in medicine* 1998; 17: 873-890.
7. Staquet M, Rozencweig M, Lee YJ, et al. Methodology for the assessment of new dichotomous diagnostic tests. *Journal of Chronic Diseases* 1981; 34: 599-610. Article. DOI: 10.1016/0021-9681(81)90059-X.
8. Brenner H. Correcting for exposure misclassification using an alloyed gold standard. *Epidemiology* 1996; 7: 406-410. Article.
