# Supplementary material for: Comparative diagnostic accuracy studies with an imperfect reference standard – a comparison of correction methods
Source: BMC Med Res Methodol. 2021 Apr 12;21:67. doi: 10.1186/s12874-021-01255-4 (PMC8040223; doi:10.1186/s12874-021-01255-4)
Supplement: Supplementary file 2 — Additional file 2. [file 12874_2021_1255_MOESM2_ESM.pdf]

Comparative diagnostic accuracy studies with an imperfect reference standard – A comparison of correction methods

Chinyereugo M. Umemneku Chikere[1*], Kevin J. Wilson[2], A. Joy Allen[3], Luke Vale[1]

[1] Population Health Science Institute, Faculty of Medical Sciences, Newcastle University

[2] School of Mathematics, Statistics and Physics, Newcastle University

[3] National Institute for Health Research, Newcastle In Vitro Diagnostics Co-operative, Newcastle University

* Corresponding author

Email: cmuc1@leicester.ac.uk (CMUC)

R-code employed to simulate the dataset, analyse generated dataset and clinical dataset

This section explains the R-Code employed to simulate the different datasets explored in this paper and to analyse the clinical datasets.

1. Calculate the cell probabilities for multinomial distribution using the fixed effect modelling approach[1, 2]

```
```{r cell probabilities}
proba<- function(pd,sRS,spRS, sIT, spIT, cova1, cova2){
#sRS and spRS are sensitivity and specificity of RS respectively
#sIT and spIt are sensitivity and specificity of IT respectively
#cova1 is the covariance term among the diseased group
#cova2 is the covariance term among the non-diseased group
#pd is the prevalence of the target condition
a<- pd*(sRS*sIT + cova1)+((1 -pd)*(1-spRS)*(1-spIT) + cova2)
  c<- pd*(sRS*(1 - sIT) - cova1) + ((1 -pd)*( 1- spRS)*spIT + cova2)
  b<- pd*((1 - sRS)*sIT - cova1) + ((1 -pd)*spRS*(1-spIT) + cova2)
  d<- pd*((1 - sRS)*(1 - sIT) + cova1) + ((1 -pd)*spRS*spIT + cova2)
prom<- c(a, c, b, d)
return(prom)
}
```
```

2. Code employed to estimate the unadjusted and corrected sensitivity and specificity of the index test.

```
```{r fun1}
cal<- function(dtab, sRS, spRS){
#dtab is the 2 by 2 matrix simulated using the multinomial distribution and the cell probability function (#1)
Np<- sum(dtab[1,1],dtab[1,2],dtab[2,1],dtab[2,2]) # total number of participants
  e<- sum(dtab[1,1], dtab[2,1]) # a+c total RS positive
  f<- sum(dtab[1,2], dtab[2,2]) # b+d total RS negative
  g<- sum(dtab[1,1], dtab[1,2]) #a+b total IT positive
  h<- sum(dtab[2,1], dtab[2,2]) # c+d # total IT negative
  prev<- e/Np # sample prevalence
  senIT <- dtab[1,1]/ e  # unadjusted sensitivity of index test
  specIT<- dtab[2,2]/f # unadjusted specificity of index test
  senbre<- (prev*sRS*senIT + (1 - prev)*(1 - spRS)*(1 - specIT))/(prev*sRS + (1 - prev)*(1 - spRS)) # Brenner corrected sensitivity
  specbre<- (prev*(1 - sRS)*(1-senIT) + (1 - prev)*(spRS)*(specIT))/(prev*(1-sRS) + (1 - prev)*spRS) # Brenner corrected specificity
  senstaq<- (g*spRS - dtab[1,2])/ (Np*(spRS - 1) + e) # Staquet et al corrected sensitivity
```
```

```
  specstaq<- (h*sRS - dtab[2,1])/(Np*sRS - e) # Staquet et al corrected specificity
  estpre<- (prev + spRS - 1)/(sRS + spRS - 1) # estimated prevalence
  result<- c(senIT, specIT, senbre, specbre, senstaq, specstaq, estpre, prev)
}
```

3. Code employed to estimate the covariance inequalities (boundary to decide the choice of covariance terms given that the index test and reference standard are conditionally dependent)

```{r covabound}
covabound<- function(sRS, spRS, sIT, spIT){
  lcovsen<- (-sRS * sIT) + max(0, sRS + sIT -1) #lower value for covariance among diseased group
  ucovsen<- min(sRS,sIT) - (sRS * sIT) #upper value of covariance among the diseased group
  lcovspec<- -spRS*spIT + max(0, spRS +spIT - 1) #lower value for covariance among non – diseased group
    ucovaspec<- min(spRS,spIT) - (spRS * spIT) #upper value of covariance among the diseased group
return (cbind(c("lower sen", "upper sen", "lower spec", "upper spec"),c(lcovsen, ucovsen, lcovspec, ucovaspec)))
}
```

4. Code to generate random samples of 2 by 2 tables under the assumption of conditional independence and conditional depedence using the possible covariance terms using the cell probabilties function.

```{r multinomial}
sim<- function(numb,n,pd,sRS,spRS, sIT, spIT, cova1, cova2){
  # n is the sample size (number of participants)
  # numb is the number of samples simulated with size n
prom<- proba(pd,sRS,spRS, sIT, spIT, cova1, cova2)
exp<- rmultinom(numb,n,prom)
tab<- list()
  for(i in 1:numb){
    tab[[i]]<- matrix(exp[,i],2, 2)
  }
return(tab)
}
```

```
### Example
set.seed(1235679)
exsim<- sim(1,100,0.9,1,1,0.8,0.7,0.05, 0.05)
```

5. Estimate the unadjusted and corrected sensitivty and specficty from numb samples simulated

```{r solve}
sol<- function(numb,n,pd,sRS,spRS, sIT, spIT, cova1, cova2){
  tabu<- sim(numb, n, pd, sRS, spRS, sIT, spIT, cova1, cova2)
  mat<- matrix(NA, numb, 8)
  for (i in 1:numb){
   mat[i,] <-  cal(tabu[[i]], sRS,spRS)
  }
  colnames(mat) <- c("Unadjsen","Unadjspec", "Sen.Brenner", "Spec.Brenner",
"Sen.Staquet", "Spec.Staquet", "EstPre", "Sam.Prev")
  tabmat<- list(tabu, mat)
  return(tabmat)
}
```

```
## Example
set.seed(1235679)
sol(10, 50, 0.3, 0.9, 0.9, 0.8, 0.7, 0,0)[[2]][,1]
```

6. Obtain the mean values of estimates, standard deviation, mean square error (MSE) and bias.

#Call up the required packages in R

```{r call}
library(gstat)
library(e1071)
library(hydroGOF)
```

Function to estimate the Mean, MSE, SD and bias of the unadjusted and corrected sensitivity and specificity of index test

```{r descriptive}
desol<- function(numb,n,pd,sRS,spRS, sIT, spIT, cova1, cova2){
  msol<- sol(numb,n,pd,sRS,spRS, sIT, spIT, cova1, cova2)[[2]]
msol1<- msol[!rowSums(!is.finite(msol)),]# remove rows with inf values or non- finite
values.
msol2<- msol1[!rowSums(msol1 > 2),] # remove rows with any value above 2.
#Values above 1 or below 0 are obtained via the Staquet et al approach.
mval<- apply(msol1, 2, mean)
sval<- apply(msol1, 2, sd)
new<- msol1
numb1<- length(msol1[,1])
```

para<- cbind(rep(sIT, numb1),rep(spIT, numb1),rep(sIT, numb1), rep(spIT, numb1),rep(sIT, numb1), rep(spIT, numb1),rep(pd, numb1),rep(pd, numb1))
msqerror<- mse(new, para)
realval<- c(sIT, spIT, sIT, spIT, sIT, spIT, pd, pd)
BiasEP<- abs(mval - realval)
MCerr<- sval/sqrt(numb)
tog<- cbind(mval,sval, msqerror, BiasEP, MCerr)
returns only the mean value, standard deviation and MSE
return(tog)
}
```
###### Simulated examples
## Imperfect test RS better than IT, IT and RS are conditionally independent
```{r example1}
set.seed(1235679)
example0<- desol(200,50,0.3, 0.9, 0.9, 0.8, 0.7, 0.00, 0.00)
example1<- desol(200,80,0.3, 0.9, 0.9, 0.8, 0.7, 0.00, 0.00)
example2<- desol(200,100,0.3,0.9, 0.9, 0.8, 0.7, 0.00, 0.00)
example3<- desol(200,120,0.3, 0.9, 0.9, 0.8, 0.7, 0.00, 0.00)
example4<- desol(200,150,0.3, 0.9, 0.9, 0.8, 0.7, 0.00, 0.00)
example5<- desol(200,180,0.3, 0.9, 0.9, 0.8, 0.7, 0.00, 0.00)
example6<- desol(200,200,0.3, 0.9, 0.9, 0.8, 0.7, 0.00, 0.00)
example7<- desol(200,250,0.3, 0.9, 0.9, 0.8, 0.7, 0.00, 0.00)
example8<- desol(200,300,0.3, 0.9, 0.9, 0.8, 0.7, 0.00, 0.00)
example9<- desol(200,350,0.3, 0.9, 0.9, 0.8, 0.7, 0.00, 0.00)
example10<- desol(200,400,0.3, 0.9, 0.9, 0.8, 0.7, 0.00, 0.00)
example11<- desol(200,500,0.3, 0.9, 0.9, 0.8, 0.7, 0.00, 0.00)
example12<- desol(200,600,0.3, 0.9, 0.9, 0.8, 0.7, 0.00, 0.00)
example13<- desol(200,700,0.3, 0.9, 0.9, 0.8, 0.7, 0.00, 0.00)
example14<- desol(200,800,0.3, 0.9, 0.9, 0.8, 0.7, 0.00, 0.00)
example15<- desol(200,900,0.3, 0.9, 0.9, 0.8, 0.7, 0.00, 0.00)
example16<- desol(200,1000,0.3, 0.9, 0.9, 0.8, 0.7, 0.00, 0.00)
```
```{r example2}
### Imperfect test RS worse than IT, IT and RS are conditionally independent
set.seed(1235679)
example0<- desol(200,50,0.3, 0.8, 0.7,0.9, 0.9,  0.00, 0.00)
example1<- desol(200,80,0.3, 0.8, 0.7,0.9, 0.9, 0.00, 0.00)
example2<- desol(200,100,0.3,0.8, 0.7,0.9, 0.9, 0.00, 0.00)
example3<- desol(200,120,0.3, 0.8, 0.7, 0.9, 0.9, 0.00, 0.00)
example4<- desol(200,150,0.3, 0.8, 0.7, 0.9, 0.9, 0.00, 0.00)
example5<- desol(200,180,0.3,0.8, 0.7, 0.9, 0.9,  0.00, 0.00)
example6<- desol(200,200,0.3,0.8, 0.7, 0.9, 0.9,  0.00, 0.00)

example7<- desol(200,250,0.3, 0.8, 0.7, 0.9, 0.9,  0.00, 0.00)
example8<- desol(200,300,0.3, 0.8, 0.7, 0.9, 0.9,  0.00, 0.00)
example9<- desol(200,350,0.3, 0.8, 0.7, 0.9, 0.9,  0.00, 0.00)
example10<- desol(200,400,0.3, 0.8, 0.7, 0.9, 0.9,  0.00, 0.00)
example11<- desol(200,500,0.3, 0.8, 0.7, 0.9, 0.9,  0.00, 0.00)
example12<- desol(200,600,0.3,0.8, 0.7,  0.9, 0.9,  0.00, 0.00)
example13<- desol(200,700,0.3,0.8, 0.7, 0.9, 0.9,  0.00, 0.00)
example14<- desol(200,800,0.3, 0.8, 0.7,0.9, 0.9,  0.00, 0.00)
example15<- desol(200,900,0.3, 0.8, 0.7,0.9, 0.9,  0.00, 0.00)
example16<- desol(200,1000,0.3,0.8, 0.7, 0.9, 0.9,  0.00, 0.00)
```

Put performance measures in a single data frame

```{r data1}
toptab<- cbind(example0[,2],example1[,2], example2[,2], example3[,2], example4[,2], example5[,2], example6[,2], example7[,2], example8[,2], example9[,2], example10[,2], example11[,2], example12[,2], example13[,2], example14[,2], example15[,2], example16[,2])
samsize<- c(50, 80,100, 120, 150, 180, 200, 250, 300, 350, 400, 500, 600, 700, 800, 900, 1000)
ttab1<- t(toptab)
ttab<- ttab1[,1:6]
colnames(ttab)<- c("sdUnadjsen","sdUnadjspec", "sdSen.Brenner", "sdSpec.Brenner", "sdSen.Staquet", "sdSpec.Staquet")

dimtab<- cbind(example0[,1],example1[,1], example2[,1], example3[,1], example4[,1], example5[,1], example6[,1], example7[,1], example8[,1], example9[,1], example10[,1], example11[,1], example12[,1], example13[,1], example14[,1], example15[,1], example16[,1])
tdimtab1<- t(dimtab)
tdimtab<- tdimtab1[,1:6]
colnames(tdimtab)<- c("meanUnadjsen","meanUnadjspec", "meanSen.Brenner", "meanSpec.Brenner", "meanSen.Staquet", "meanSpec.Staquet")

msqtab<- cbind(example0[,3],example1[,3], example2[,3], example3[,3], example4[,3], example5[,3], example6[,3], example7[,3], example8[,3], example9[,3], example10[,3], example11[,3], example12[,3], example13[,3], example14[,3], example15[,3], example16[,3])
tmsqtab1<- t(msqtab)
tmsqtab<- tmsqtab1[,1:6]
colnames(tmsqtab)<- c("msqUnadjsen","msqUnadjspec", "msqSen.Brenner", "msqSpec.Brenner", "msqSen.Staquet", "msqSpec.Staquet")

biastab<- cbind(example0[,4],example1[,4], example2[,4], example3[,4], example4[,4], example5[,4], example6[,4], example7[,4], example8[,4], example9[,4], example10[,4],

~~~
example11[,4], example12[,4], example13[,4], example14[,4], example15[,4], example16[,4])
tbtab1<- t(biastab)
tbtab<- tbtab1[,1:6]
colnames(tbtab)<- c("biasUnadjsen","biasUnadjspec", "biaSen.Brenner", "biaSpec.Brenner", "biaSen.Staquet", "biaSpec.Staquet")

MCerrtab<- cbind(example0[,5],example1[,5], example2[,5], example3[,5], example4[,5], example5[,5], example6[,5], example7[,5], example8[,5], example9[,5], example10[,5], example11[,5], example12[,5], example13[,5], example14[,5], example15[,5], example16[,5])
MCtab1<- t(MCerrtab)
MCtab<- MCtab1[,1:6]
colnames(MCtab)<- c("MCerrUnadjsen","MCerrUnadjspec", "MCerrSen.Brenner", "MCerrSpec.Brenner", "MCerrSen.Staquet", "MCerrSpec.Staquet")

samtab<- round(data.frame(samsize, ttab,tdimtab, tmsqtab, tbtab, MCtab),4)
```
~~~

Other possible variations or conditions can be explored by changing the values of the sensitivities and specificities of IT and or RS and prevalence.

7. Plot the unadjusted and corrected mean sensitivty and specificty of IT alongside the SD, Bias and MSE

~~~
## call up required R packages
```{r library}
library(dplyr)
library(tidyr)
library(ggplot2)
library(reshape2)
library(gridExtra)
```

##### plot performance measures against sample size
```{r plot2}
My_Theme = theme(axis.title.x = element_text(size = 16),axis.text.x = element_text(size = 14),axis.title.y = element_text(size = 16), axis.text.y = element_text(size = 14), legend.title=element_text(size=12),legend.text=element_text(size=12))

df <- melt(samtab[,c("samsize","sdUnadjsen", "sdSen.Brenner", "sdSen.Staquet")], id="samsize")
pg <- df    # Copy data into new data frame
# Rename the column and the values in the factor
levels(pg$variable)[levels(pg$variable)=="sdUnadjsen"] <- "Unadjusted"
levels(pg$variable)[levels(pg$variable)=="sdSen.Brenner"] <- "Brenner"
~~~

levels(pg$variable)[levels(pg$variable)=="sdSen.Staquet"] <- "Staquet"
names(pg)[names(pg)=="variable"] <- "Standard.Error"

p<- ggplot(pg, aes(x=samsize, y=value, col= Standard.Error)) + geom_line() + labs(x ="Sample size", y = "SE sensitivity")   + geom_line(size = 1) + coord_cartesian(ylim=c(0.0,0.3))+ My_Theme
#+ geom_hline(yintercept=0.9, linetype="dashed", color = "yellow", size = 2)

specificity
df1 <- melt(samtab[,c("samsize","sdUnadjspec", "sdSpec.Brenner", "sdSpec.Staquet")], id="samsize")
pg1 <- df1   # Copy data into new data frame
# Rename the column and the values in the factor
levels(pg1$variable)[levels(pg1$variable)=="sdUnadjspec"] <- "Unadjusted"
levels(pg1$variable)[levels(pg1$variable)=="sdSpec.Brenner"] <- "Brenner"
levels(pg1$variable)[levels(pg1$variable)=="sdSpec.Staquet"] <- "Staquet"
names(pg1)[names(pg1)=="variable"] <- "Standard.Error"

p1<- ggplot(pg1, aes(x=samsize, y=value, col= Standard.Error)) + geom_line() + labs(x ="Sample size", y = "SE specificity") + geom_line(size = 1) + coord_cartesian(ylim=c(0.0,0.07))+ My_Theme
#+ geom_hline(yintercept=0.9, linetype="dashed", color = "yellow", size = 2)

put both plot as one
grid.arrange(p, p1, nrow=2)
```

8. Estimate the mean sensitivity and specificity of IT at varying prevalences

## Estimate only the mean sensitivity and specificity of IT
```{r meansol}
meansol<- function(numb,n,pd,sRS,spRS, sIT, spIT, cova1, cova2){
  msol<- sol(numb,n,pd,sRS,spRS, sIT, spIT, cova1, cova2)[[2]]
  msol1<- msol[!rowSums(!is.finite(msol)),]# remove rows with inf values or non- finite values.
  msol2<- msol1[!rowSums(msol1 > 2),] # exclude rows greater than 2
  msol3<- msol2[!rowSums(msol2 < 0),] #exclude rows less than zero
meanval<- apply(msol3, 2, mean)
return(meanval)
}
```

Code that estimates the mean values of the estimator at different prevalence
```{r soldiff}
soldiff<- function (z,numb,n,sRS,spRS, sIT, spIT, cova1, cova2){

````
  pd<- seq(0.00, 1, length.out = z)
  top<- list()
  for(i in 1:z){
    top[[i]]<- meansol(numb,n,pd[i],sRS,spRS,sIT,spIT, cova1, cova2)
  }
  tim<- matrix(NA,z, 8)
  for(i in 1:z){
    tim[i,]<- top[[i]]
  }
  ss<- rep(n, z)
 colnames(tim) <- c("Unadjsen","Unadjspec", "Sen.Brenner", "Spec.Brenner",
"Sen.Staquet", "Spec.Staquet", "EstPre", "Sam.Prev")
 timpd<- cbind(pd, tim,ss)#data.frame
return(timpd)
}
```
### Simulate 1000 participants, 200 multiple samples, 100 prevelances. RS is better than IT and RS is imperfect
```{r data}
set.seed(1235679)
preout<- soldiff(100,200,1000, 0.9, 0.9, 0.8, 0.8, 0.00, 0.00)
preout<- data.frame(preout)
```

plot the mean sensitivity and specificity
```{r plot2}
My_Theme = theme(axis.title.x = element_text(size = 16),axis.text.x = element_text(size = 14),axis.title.y = element_text(size = 16), axis.text.y = element_text(size = 14), legend.title=element_text(size=12),legend.text=element_text(size=12))

df <- melt(preout[,c("pd","Unadjsen", "Sen.Brenner", "Sen.Staquet")], id="pd")
pg <- df    # Copy data into new data frame
# Rename the column and the values in the factor
levels(pg$variable)[levels(pg$variable)=="Unadjsen"] <- "Unadjusted"
levels(pg$variable)[levels(pg$variable)=="Sen.Brenner"] <- "Brenner"
levels(pg$variable)[levels(pg$variable)=="Sen.Staquet"] <- "Staquet"
names(pg)[names(pg)=="variable"]  <- "Mean"

p<- ggplot(pg, aes(x=pd, y=value, col= Mean)) + geom_line()+ geom_point() + labs(x ="Prevelance", y = "Mean sensitivity")   + geom_line(size = 1) + coord_cartesian(ylim=c(0.2,1))+ My_Theme + geom_hline(yintercept=0.8, linetype="dashed", color = "yellow", size = 2)

##### specificity
````

```
df1 <- melt(preout[,c("pd","Unadjspec", "Spec.Brenner", "Spec.Staquet")], id="pd")
pg1 <- df1   # Copy data into new data frame
# Rename the column and the values in the factor
levels(pg1$variable)[levels(pg1$variable)=="Unadjspec"] <- "Unadjusted"
levels(pg1$variable)[levels(pg1$variable)=="Spec.Brenner"] <- "Brenner"
levels(pg1$variable)[levels(pg1$variable)=="Spec.Staquet"] <- "Staquet"
names(pg1)[names(pg1)=="variable"]  <- "Mean"

p1<- ggplot(pg1, aes(x=pd, y=value, col= Mean)) + geom_line() + geom_point() + labs(x ="Prevelance", y = "Mean specificity")  + geom_line(size = 1) + coord_cartesian(ylim=c(0.4,1.2))+ My_Theme + geom_hline(yintercept=0.8, linetype="dashed", color = "yellow", size = 2)


### put both plot as one
grid.arrange(p, p1, nrow=2)
```

9. Estimate the mean corrected and unadjusted sensitivty and specificty of IT assuming sensitivity of RS (or specificty of RS) varies from 0 to 1. Unlike the function in 6 and 8 above the sensitivty of RS and IT, and the specificity of RS and IT are fixed. This allows more possible combinations to examine how the corrections method perform.

    #### estimate mean sensitivity and specificity by fixing one of these parameters – sRS, spRS, sIT, spIT- and varying the others

```{r soldiff1}
soldiff1<- function (z,pd,numb,n,sRS, spRS, sIT, cova1, cova2){
##in this stated function the spIT is varied and the others are fixed
## to vary another parameter change it appropraitely
  spIT<- seq(0, 1, length.out = z)
  top<- list()
  for(i in 1:z){
    top[[i]]<- meansol(numb,n,pd,sRS,spRS,sIT,spIT[i], cova1, cova2)
  }
  tim<- matrix(NA,z, 8)
  for(i in 1:z){
    tim[i,]<- top[[i]]
  }
  ss<- rep(n, z)
 colnames(tim) <- c("Unadjsen","Unadjspec", "Sen.Brenner", "Spec.Brenner", "Sen.Staquet", "Spec.Staquet", "EstPre", "Sam.Prev")
 timpd<- cbind(spIT, tim,ss)#data.frame
return(timpd)
}
```

````
### explore1000 partcipants, 200 multiple samples
```{r explore}
set.seed(1235679)
preout<- soldiff1(100,0.3,200,1000, 0.9, 0.9, 0.8, 0.00, 0.00)
preout<- data.frame(preout)
```
##### plot mean sensitivity and specificity
```{r plot2}
My_Theme = theme(axis.title.x = element_text(size = 16),axis.text.x = element_text(size = 14),axis.title.y = element_text(size = 16), axis.text.y = element_text(size = 14), legend.title=element_text(size=12),legend.text=element_text(size=12))

df <- melt(preout[,c("spIT","Unadjsen", "Sen.Brenner", "Sen.Staquet")], id="spIT")
pg <- df    # Copy data into new data frame
# Rename the column and the values in the factor
levels(pg$variable)[levels(pg$variable)=="Unadjsen"] <- "Unadjusted"
levels(pg$variable)[levels(pg$variable)=="Sen.Brenner"] <- "Brenner"
levels(pg$variable)[levels(pg$variable)=="Sen.Staquet"] <- "Staquet"
names(pg)[names(pg)=="variable"]  <- "Mean"

p<- ggplot(pg, aes(x=spIT, y=value, col= Mean)) + geom_line()+ geom_point() + labs(x ="Specificity IT", y = "Mean sensitivity")     + geom_line(size = 1) + coord_cartesian(ylim=c(0.4,1))+ My_Theme + geom_hline(yintercept=0.8, linetype="dashed", color = "yellow", size = 2)

##### specificity
df1 <- melt(preout[,c("spIT","Unadjspec", "Spec.Brenner", "Spec.Staquet")], id="spIT")
pg1 <- df1   # Copy data into new data frame
# Rename the column and the values in the factor
levels(pg1$variable)[levels(pg1$variable)=="Unadjspec"] <- "Unadjusted"
levels(pg1$variable)[levels(pg1$variable)=="Spec.Brenner"] <- "Brenner"
levels(pg1$variable)[levels(pg1$variable)=="Spec.Staquet"] <- "Staquet"
names(pg1)[names(pg1)=="variable"]  <- "Mean"

p1<- ggplot(pg1, aes(x=spIT, y=value, col= Mean)) + geom_line() + geom_point() + labs(x ="Specificity IT", y = "Mean specificity")    + geom_line(size = 1) + coord_cartesian(ylim=c(0,1))+ My_Theme +geom_abline(intercept = 0,slope = 1, color="yellow", linetype="dashed", size=2)
#+ geom_hline(yintercept=0.8, linetype="dashed", color = "yellow", size = 2)

### put both plot as one
grid.arrange(p, p1, nrow=2)
```
````

10. Code to estimate the sensitivty and specificty of IT which include the Brenner second estimators for positively correlated IT and RS. This pair of estimators is explored in Appendix File 4.

```
```{r Brenner positive fun}
calpos<- function(dtab, sRS, spRS){
Np<- sum(dtab[1,1],dtab[1,2],dtab[2,1],dtab[2,2]) # total number of participants
  e<- sum(dtab[1,1], dtab[2,1]) # a+c total RS positive
  f<- sum(dtab[1,2], dtab[2,2]) # b+d total RS negative
  g<- sum(dtab[1,1], dtab[1,2]) #a+b total IT positive
  h<- sum(dtab[2,1], dtab[2,2]) # c+d # total IT negative
  prev<- e/Np # prevalence of the diseased in sample of study
  senIT <- dtab[1,1]/ e  # sensitivity of index test unadjusted
  specIT<- dtab[2,2]/f # specificity of index test unadjusted
  senpos<- (prev * senIT +(1 - prev)*(1 - spRS))/(prev*sRS + (1 - prev)*(1 - spRS)) # corrected sensitivity of IT using the positively correlated pair of estimator by Brenner
  specpos<- (prev * ( 1 - sRS) +(1 - prev)*specIT)/(prev*( 1 - sRS) + (1 - prev)*spRS) # corrected sensitivity of IT using the positively correlated pair of estimator by Brenner
  senbre<- (prev*sRS*senIT + (1 - prev)*(1 - spRS)*(1 - specIT))/(prev*sRS + (1 - prev)*(1 - spRS))
  specbre<- (prev*(1 - sRS)*(1-senIT) + (1 - prev)*(spRS)*(specIT))/(prev*(1-sRS) + (1 - prev)*spRS)
  senstaq<- (g*spRS - dtab[1,2])/ (Np*(spRS - 1) + e)
  specstaq<- (h*sRS - dtab[2,1])/(Np*sRS - e)
  estpre<- (prev + spRS - 1)/(sRS + spRS - 1)
  return( c(senpos, specpos, senIT, specIT, senbre, specbre, senstaq, specstaq))
}
```
```

```
### Code to generate random samples of estimate the mean values
```{r Brenner positive}
solpos<- function(numb,n,pd,sRS,spRS, sIT, spIT, cova1, cova2){
  tabu<- sim(numb, n, pd, sRS, spRS, sIT, spIT, cova1, cova2)
  mat<- matrix(NA, numb, 8)
  for (i in 1:numb){
   mat[i,] <-  calpos(tabu[[i]], sRS,spRS)
  }
  colnames(mat) <- c("Sen.pos.Bre", "Spec.pos.Bre", "Unadjsen", "UnadjSpec","Sen.Brenner","Spec.Brenner","Sen.Staquet", "Spec.Staquet")
  meanmat<- apply(mat, 2, mean)
  #tabmat<- list(tabu, mat)
  return(meanmat)
}
```
```

Code to estimate the mean values at different prevelances and result

````
```{r soldiff}
solposdiff<- function (z,numb,n,sRS,spRS, sIT, spIT, cova1, cova2){
  pd<- seq(0, 1, length.out = z)
  top<- list()
  for(i in 1:z){
    top[[i]]<- solpos(numb,n,pd[i],sRS,spRS,sIT,spIT, cova1, cova2)
  }
  ss<- rep(n, z)
  tim<- matrix(NA,z, 8)
  for(i in 1:z){
    tim[i,]<- top[[i]]
  }
 colnames(tim) <- c("Sen.pos.Bre", "Spec.pos.Bre", "Unadjsen",
"UnadjSpec","Sen.Brenner", "Spec.Brenner","Sen.Staquet", "Spec.Staquet")
 timpd<- data.frame(pd, tim, ss)
return(timpd)
}
```

#### simulate dataset with IT and RS conditionally dependent and covariance terms
among the disease and non-diseased group are 0.05.
```{r solposdiff}
set.seed(1235679)
preout<- solposdiff(100,200,1000,0.9,0.9,0.8,0.8,0.05,0.05)
preout<- data.frame(preout)
```


### plot the mean values against the prevalences
```{r plot2}
My_Theme = theme(axis.title.x = element_text(size = 16),axis.text.x = element_text(size
= 14),axis.title.y = element_text(size = 16), axis.text.y = element_text(size = 14),
legend.title=element_text(size=12),legend.text=element_text(size=12))

df <- melt(preout[,c("pd","Unadjsen", "Sen.Brenner", "Sen.Staquet", "Sen.pos.Bre")],
id="pd")
pg <- df    # Copy data into new data frame
# Rename the column and the values in the factor
levels(pg$variable)[levels(pg$variable)=="Unadjsen"] <- "Unadjusted"
levels(pg$variable)[levels(pg$variable)=="Sen.Brenner"] <- "Brenner"
levels(pg$variable)[levels(pg$variable)=="Sen.Staquet"] <- "Staquet"
levels(pg$variable)[levels(pg$variable)=="Sen.pos.Bre"] <- "BrennerPos"
names(pg)[names(pg)=="variable"]  <- "Mean"
````
````

````
p<- ggplot(pg, aes(x=pd, y=value, col= Mean)) + geom_line()+ geom_point() + labs(x
="Prevelance", y = "Mean sensitivity")   + geom_line(size = 1) +
coord_cartesian(ylim=c(0.0,1))+ My_Theme + geom_hline(yintercept=0.8,
linetype="dashed", color = "yellow", size = 2)

##### specificity
df1 <- melt(preout[,c("pd","UnadjSpec", "Spec.Brenner", "Spec.Staquet",
"Spec.pos.Bre")], id="pd")
pg1 <- df1   # Copy data into new data frame
# Rename the column and the values in the factor
levels(pg1$variable)[levels(pg1$variable)=="UnadjSpec"] <- "Unadjusted"
levels(pg1$variable)[levels(pg1$variable)=="Spec.Brenner"] <- "Brenner"
levels(pg1$variable)[levels(pg1$variable)=="Spec.Staquet"] <- "Staquet"
levels(pg1$variable)[levels(pg1$variable)=="Spec.pos.Bre"] <- "BrennerPos"
names(pg1)[names(pg1)=="variable"]  <- "Mean"

p1<- ggplot(pg1, aes(x=pd, y=value, col= Mean)) + geom_line() + geom_point() + labs(x
="Prevelance", y = "Mean specificity")  + geom_line(size = 1) +
coord_cartesian(ylim=c(0.0,1))+ My_Theme + geom_hline(yintercept=0.8,
linetype="dashed", color = "yellow", size = 2)


### put both plot as one
grid.arrange(p, p1, nrow=2)
```
````

11. Calculate the sensitivity and specificity of the clinical dataset

````
```{r clinical1}
### use the cal function and put the sRS and spRS appropriately.
## Matos et al NC dataset
tabLF1<- matrix(c(241, 110,6,26), 2, 2) # matrix for LFpen
tabFC1<- matrix(c(156, 195,2,30), 2, 2) # matrix for FC
tiLF1<- cal(tabLF1, 0.796,0.799) # estimates for LFpen
tiFC1<- cal(tabFC1, 0.796,0.799) # estimates for FC
tiLF1
tiFC1
```

## D3 classification
```{r clinical2}
tabLF1<- matrix(c(20, 1,45,341), 2, 2) # matrix for LFpen
tabFC1<- matrix(c(21, 0,38,348), 2, 2) # matrix for FC
tiLF1<- cal(tabLF1, 0.786, 0.995) # estimates for LFpen
tiFC1<- cal(tabFC1, 0.786, 0.995) # estimates for FC
tiLF1
````

```
tiFC1
```

12. Calculate the 95% confidence interval of clinical dataset using the Wilson score interval

```
# Code Wilson score interval
```{r wilson}
wilfun<- function(p, n){
  z<- qnorm(1-0.05/2)
  rt<- 1/(1 + (z^2 /n))
  rt1<- p + (z^2/(2*n))
  rtp<- rt*rt1
  vart<- ((p*(1 - p))/n) + ((z^2)/(4*(n^2)))
  zvar<- (z/(1 + ((z^2)/n))) * sqrt(vart)
  LL<- rtp-zvar
  UL<- rtp+zvar
  return(c(LL, UL))
}
```

calculate 95%CI Mathew dataset
```{r cal}
wilsu<- wilfun(0.65, 62) # sensitivity unadjusted
wilsb<- wilfun(0.5,62 ) # Brenner corrected sensitivity
wilss<- wilfun(0.89,62)# Staquet corrected sensitivity
wilpu<- wilfun(0.89, 199)# unadjusted specificity
wilpb<- wilfun(0.85, 199) # Brenner corrected specificity
wilps<- wilfun(0.96, 199) # Staquet et al corrected specificity
wilsu
wilsb
wilss
wilpu
wilpb
wilps
```

calculate 95% CI of the sensitivity of LFpen for NC detection
```{r cal}
wilu<- se(351, 32, 0.81, 0.69)
wilb<- se(351, 32,0.44, 0.68)
wils<- se(351, 32, 0.04, 0.70)
wilu
wilb
wils
```
```

```

## calculate 95% CI of the sensitivity of FC for NC detection
```{r cal}
wilu<- se(351, 32, 0.91, 0.44)
wilb<- se(351, 32, 0.65, 0.44)
wils<- se(351, 32, 0.36, 0.45)
wilu
wilb
wils
```

calculate the 95% CI for unadjusted specificity FC- D3 dataset
```{r test}
install.packages("DescTools")
library("DescTools")
BinomCI(348, 386, 0.95, sides = "two.sided", method = "wilson")

## References

1. Wang Z, Dendukuri N, Zar HJ, et al. Modeling conditional dependence among multiple diagnostic tests. *Statistics in medicine* 2017; 36: 4843-4859.

2. Vacek PM. The effect of conditional dependence on the evaluation of diagnostic tests. *Biometrics* 1985; 41: 959-968.
