# Supplementary material for: Comparative diagnostic accuracy studies with an imperfect reference standard – a comparison of correction methods
Source: BMC Med Res Methodol. 2021 Apr 12;21:67. doi: 10.1186/s12874-021-01255-4 (PMC8040223; doi:10.1186/s12874-021-01255-4)
Supplement: Supplementary file 3 — Additional file 3. [file 12874_2021_1255_MOESM3_ESM.pdf]

Comparative diagnostic accuracy studies with an imperfect reference standard – A comparison of correction methods

Chinyereugo M. Umemneku Chikere[1*], Kevin J. Wilson[2], A. Joy Allen[3], Luke Vale[1]

[1] Population Health Science Institute, Faculty of Medical Sciences, Newcastle University

[2] School of Mathematics, Statistics and Physics, Newcastle University

[3] National Institute for Health Research, Newcastle In Vitro Diagnostics Co-operative, Newcastle University

* Corresponding author

Email: cmuc1@leicester.ac.uk (CMUC)

Conditions for obtaining illogical estimates using Staquet et al[1] approach

The Staquet et al[1] correction method is explored algebraically to understand the conditions for obtaining illogical estimates. Illogical estimates are estimates (sensitivity, specificity and prevalence) that are outside [0, 1].

Table S1: Table of added Notation

NPV	Negative predictive value
PPV	Positive predictive value
$rNPV$	Relative negative predictive value
$rPPV$	Relative positive predictive value
$rNPV'$	Complement of relative negative predictive value $(1 - rNPV)$
$rPPV'$	Complement of relative positive predictive value $(1 - rPPV)$

1.1. Illogical estimates for prevalence

Algebraically, illogical estimate (greater than one) is obtained for the estimated prevalence if:

$$N(Sp_{RS} - 1) + e > N(Sn_{RS} + Sp_{RS} - 1)$$

$$NSp_{RS} - N + e > NSn_{RS} + NSp_{RS} - N$$

$$e > NSn_{RS}$$

$$Sn_{RS} < \frac{e}{N} = Prr \qquad Condition\ (1)$$

1.2. Illogical estimates for sensitivity and specificity

Algebraically, illogical estimates are obtained for the sensitivity of the IT via the Staquet et al[1] approach if:

$$Sp_{RS} < \frac{d}{h} = rNPV \qquad Condition\ (2)$$

$$Sp_{RS} < \frac{b}{g} = rPPV' \quad \textbf{and} \quad Sp_{RS} > \frac{f}{N} = 1 - Prr = Prr' \qquad Condition\ (3a)$$

$$Sp_{RS} > \frac{b}{g} = rPPV' \quad \textbf{and} \quad Sp_{RS} < \frac{f}{N} = 1 - Prr = Prr' \qquad Condition\ (3b)$$

Condition (2) produces an estimated corrected sensitivity whose absolute value is greater than one and condition (3) produces a negative estimate that is estimate less than zero.

Similarly, illogical estimates are obtained for the specificity of IT if:

$$Sn_{RS} < \frac{a}{g} = rPPV \qquad Condition\ (4)$$

$$Sn_{RS} < \frac{c}{h} = rNPV' \quad \text{and} \quad Sn_{RS} > \frac{e}{N} = Prr \qquad Condition\ (5a)$$

$$Sn_{RS} > \frac{c}{h} = rNPV' \quad \text{and} \quad Sn_{R} < \frac{e}{N} = Prr \qquad Condition\ (5b)$$

Condition (4) produces an estimated corrected specificity whose absolute value is greater than one, and condition (5) produces negative estimates.

The $\boldsymbol{rNPV}$ refers to the ***"relative negative predictive value"***. It is the proportion of participants with negative results in both the IT and RS divided by total number of participants with a negative IT result. It is termed ***relative*** because it is obtained in

relation to the RS which is imperfect. If the RS was a gold standard, it would be called the negative predictive value (NPV). Therefore, the complement of $rNPV$ ($rNPV'$), is estimated as:

$$1 - rNPV = rNPV' = 1 - \frac{d}{h} = \frac{c}{h}$$

The ***"relative positive predictive value (rPPV)"*** is the proportion of participants with positive test results in both the IT and RS divided by the total number of participants with a positive IT result.

A table showing the number of illogical results obtained from the Staquet et al[1] approach using multiple (200) samples of varying sizes is presented in Table S2.

Table S2: The Number of illogical results obtained using the Staquet et l correction method on 200 simulated samples of various sizes

Sample size	Number of samples producing illogical sensitivity and/or specificity at different prevalence						
	**Prevalence**						
	**0.1**	**0.2**	**0.3**	**0.5**	**0.7**	**0.85**	**0.95**
50	64	33	0	3	8	42	57
80	55	26	0	0	0	27	65
100	49	23	0	0	3	18	53
120	48	13	0	0	0	12	64
150	52	11	0	0	0	6	54
200	44	13	0	0	0	4	58
250	36	10	0	0	0	3	42
300	27	6	0	0	0	3	46
350	22	0	0	0	0	3	46
400	22	2	0	0	0	0	46
500	15	0	0	0	0	0	38
700	14	0	0	0	0	0	26
1000	2	0	0	0	0	0	11

1.3. Assessing the clinical datasets for possibility of obtaining Illogical estimates

The Mathews et al[2] dataset (Table 3 in the paper) was assessed to ascertain if illogical estimates could be obtained via the Staquet et al[1] approach, the statistics below were estimated:

$$rPPV = 0.645; \quad rNPV = 0.889; \quad rPPV' = 0.355; \quad rNPV' = 0.111; \quad Prr = 0.23$$

The sensitivity of the RS (0.74) is greater than the sample prevalence (0.23), hence, obtaining illogical prevalence is unlikely. In addition, the sensitivity of RS is greater than the rPPV (0.645), the $rNPV'$ (0.111) and the sample prevalence (0.23); therefore, obtaining an illogical sensitivity via Staquet et al[1] approach is unlikely. The specificity of the RS (0.91) is greater than the rNPV (0.889), $rPPV'$ (0.355) and Prr' (0.77). Thus, an illogical specificity estimate will not be obtained using the Staquet et al approach. In summary, none of the conditions for obtaining illogical estimates were fulfilled in this dataset.

The first clinical dataset from Matos et al[3] (**Error! Reference source not found.**) was assessed for the possibility of obtaining illogical estimates and the following statistics were calculated:

NC, LFpen Examiner 1

$$rPPV = 0.975\,; \quad rPPV' = 0.025; \quad rNPV' = 0.809; \quad rNPV = 0.191; \quad Prr = 0.916$$

NC, FC Examiner 1

$$rPPV = 0.981\,; \quad rPPV' = 0.019; \quad rNPV' = 0.871; \quad rNPV = 0.129; \quad Prr = 0.916$$

The sensitivity of the RS (0.796) is less than the sample prevalence (0.92), hence, there is a likelihood of obtaining illogical estimated prevalence. The estimated prevalence is 1.2 (which is illogical). The specificity of visual inspection (0.799) is greater than the rNPV (0.191 or 0.129). It is also greater than the complement of the rPPV ($rPPV' = 0.025$) and the complement of the sample prevalence ($Prr' = 1 - Prr = 0.004$). Thus, obtaining illogical sensitivity for the index tests (LFpen and FC) are unlikely. The sensitivity of visual inspection (0.796) is less than the rPPV (0.975 or 0.981) indicating the likelihood of obtaining illogical estimates for the specificities of FC and LFpen whose absolute value is greater than one. The sensitivity of the RS is also less than the sample prevalence (0.916) and less than the complement of the relative NPV (0.871 for FC, and 0.809 for LFpen). In summary, condition (1) and condition (3a) was fulfilled in this dataset. Illogical estimated prevalence was obtained but the estimated specificities are logical (that is within [0, 1]).

The second clinical dataset from Matos et al[3] (**Error! Reference source not found.**) was assessed to ascertain of obtaining illogical results and the following statistics are calculated.

D3, LFpen Examiner 1

$$rPPV = 0.308; \quad rPPV' = 0.692; \quad rNPV' = 0.003; \quad rNPV = 0.997; \quad Prr = 0.052$$

D3, FC Examiner 1

$$rPPV = 0.356; \quad rPPV' = 0.644; \quad rNPV' = 0; \quad rNPV = 1; \quad Prr = 0.052$$

The sensitivity of the RS (0.786) is greater than the sample prevalence (0.052). Hence, obtaining illogical estimated prevalence is unlikely. The sensitivity of the RS (0.786) is

also greater than the rPPV (0.31 or 0.36), and the complement of the rNPV (0). Therefore, the likelihood of obtaining illogical specificities for LFpen and FC are unlikely. The specificity of visual inspection (0.995) is less than the rNPV (1 for FC and 0.997 for LFpen). It is also greater than the complement of the rPPV ($rPPV' = 0.025$) and the complement of the prevalence ($Prr' = 0.95$). Thus, obtaining illogical sensitivity estimates for the index tests (LFpen and FC) is likely as the condition (2) is met. In summary, illogical estimated sensitivity was obtained for the index tests (1.04 and 1.09).

References

1. Staquet M, Rozencweig M, Lee YJ, et al. Methodology for the assessment of new dichotomous diagnostic tests. *Journal of Chronic Diseases* 1981; 34: 599-610. Article. DOI: 10.1016/0021-9681(81)90059-X.
2. Mathews WC, Cachay ER, Caperna J, et al. Estimating the accuracy of anal cytology in the presence of an imperfect reference standard. *PLoS ONE* 2010; 5. Article. DOI: 10.1371/journal.pone.0012284.
3. Matos RN, T. F.; Braga, M. M.; Siqueira, W. L.; Duarte, D. A.; Mendes, F. M. Clinical performance of two fluorescence-based methods in detecting occlusal caries lesions in primary teeth. *Caries Research* 2011; 45: 294-302. Article. DOI: 10.1159/000328673.
