# Supplementary material for: Comparative diagnostic accuracy studies with an imperfect reference standard – a comparison of correction methods
Source: BMC Med Res Methodol. 2021 Apr 12;21:67. doi: 10.1186/s12874-021-01255-4 (PMC8040223; doi:10.1186/s12874-021-01255-4)
Supplement: Supplementary file 4 — Additional file 4. [file 12874_2021_1255_MOESM4_ESM.pdf]

Comparative diagnostic accuracy studies with an imperfect reference standard – A comparison of correction methods

Chinyereugo M. Umemneku Chikere[1*], Kevin J. Wilson[2], A. Joy Allen[3], Luke Vale[1]

[1] Population Health Science Institute, Faculty of Medical Sciences, Newcastle University

[2] School of Mathematics, Statistics and Physics, Newcastle University

[3] National Institute for Health Research, Newcastle In Vitro Diagnostics Co-operative, Newcastle University

* Corresponding author

Email: cmuc1@leicester.ac.uk (CMUC)

Comparison of the correction methods under the assumption that the index test and the reference standard are conditionally dependent and the reference standard is imperfect

The simulated true values for the sensitivity and specificity of the reference standard (RS) are both 0.9. The sensitivity and specificity of the index test (IT) are both 0.8. Following the inequality constraints[1, 2], the bounded value of the covariance terms among the diseased group (cov_d) are:

$$-Sn_R \times Sn_T + \max(0, Sn_R + Sn_T - 1) \leq \varphi_{1,1|1} \leq \min(Sn_R, Sn_T) - (Sn_R \times Sn_T)$$

$$\Rightarrow -0.02 \leq \varphi_{1,1|1} \leq 0.08$$

and among the covariance bound among the non-diseased group (cov_{nd}) is:

$$-Sp_R \times Sp_T + \max(0, Sp_R + Sp_T - 1) \leq \varphi_{0,0|0} \leq \min(Sp_R, Sp_T) - (Sp_R \times Sp_T)$$

$$\Rightarrow -0.02 \leq \varphi_{0,0|0} \leq 0.08$$

The closer the covariance term to zero, the less significant the covariance term.

In addition to the Brenner correction method already discussed in the paper which is used when the IT and the RS are conditionally independent, Brenner[3] proposed another correction method to correct the sensitivity and specificity of the IT when the IT and the RS are positively correlated. The second pair of estimators is:

$$Sn_{cor}^{B2} = \frac{Prr \times Sn_{IT} + (1 - Prr) \times (1 - Sp_{RS})}{Prr \times Sn_{RS} + (1 - Prr) \times (1 - Sp_{RS})}$$

$$Sp_{cor}^{B2} = \frac{Prr \times (1 - Sn_{RS}) + (1 - Prr) \times Sp_{IT}}{Prr \times (1 - Sn_{RS}) + (1 - Prr) \times Sp_{RS}}$$

When the reference standard is perfect the corrected sensitivity and specificity of the index test are the same as the unadjusted sensitivity and specificity.

$$Sn_{cor}^{B2} = \frac{Prr \times Sn_{IT} + (1 - Prr) \times (1 - Sp_{RS})}{Prr \times Sn_{RS} + (1 - Prr) \times (1 - Sp_{RS})} = \frac{Prr \times Sn_{IT}}{Prr} = Sn_{IT}$$

$$Sp_{cor}^{B2} = \frac{Prr \times (1 - Sn_{RS}) + (1 - Prr) \times Sp_{IT}}{Prr \times (1 - Sn_{RS}) + (1 - Prr) \times Sp_{RS}} = \frac{(1 - Prr) \times Sp_{IT}}{(1 - Prr)} = Sp_{IT}$$

To investigate the performance of these correction methods assuming that the tests are conditionally independent or dependent, 200 samples of 1000 participants were simulated at 100 different prevalence values (from 0 to 1) using the multinomial distribution.

Firstly, the second pair of estimators proposed by Brenner (denoted as BrennerPos) was investigated under the assumption that the IT and RS are conditionally independent alongside the classical approach, the Staquet et al[4] correction method and the Brenner correction method (already discussed in the paper). The mean unadjusted and corrected sensitivities and specificities are reported in Figure 1. The yellow dashed lines on Figure 1 and Figure 2 are the simulated true values for the sensitivities and specificities of the index test.

Figure 1: The mean of the unadjusted and corrected sensitivity and specificity of the index test under varying prevalence.

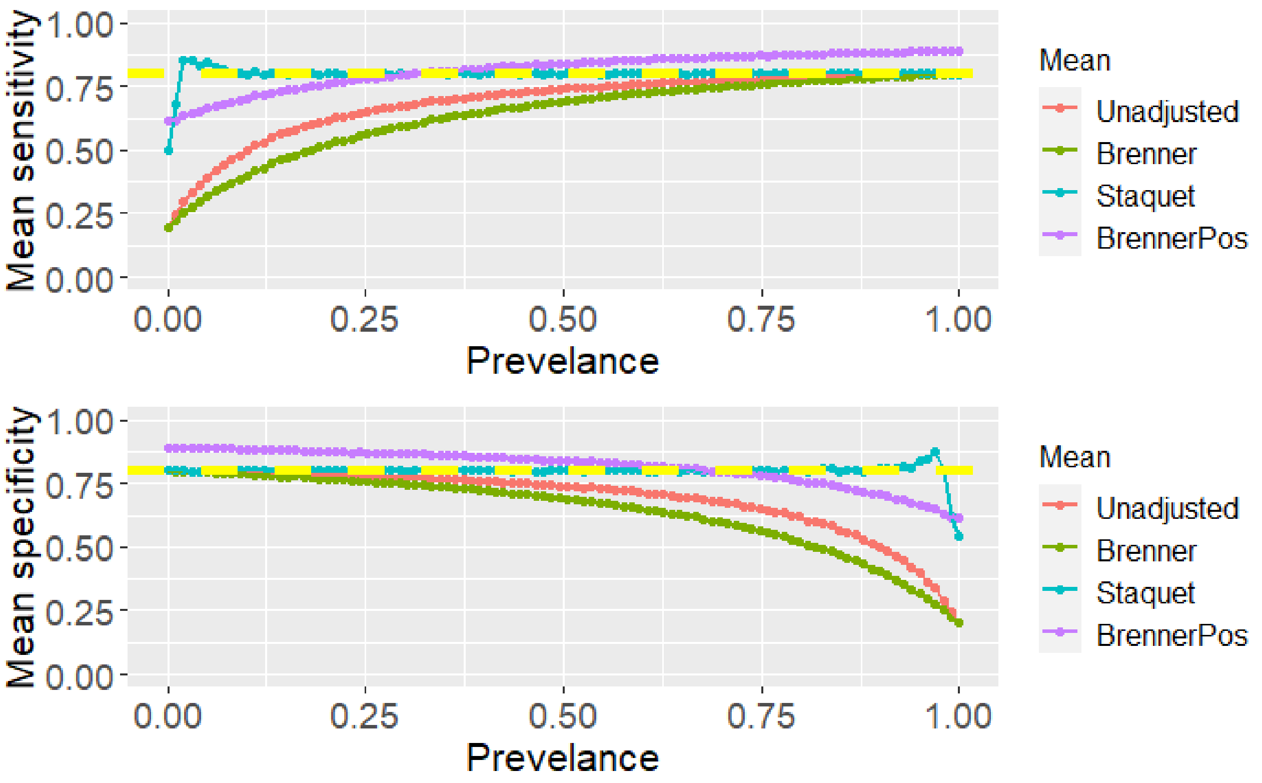

Following Figure 1, the Staquet et al[4] correction method outperforms the two estimators proposed by Brenner[3]. However, at very high and low prevalences, illogical estimates could be obtained via Staquet et al[4] correction method. As explained in the paper, some illogical points were excluded in the plot as the plot cannot capture those points.

Secondly, the correction methods were explored under the assumption that the IT and RS are conditionally dependent. The different scenarios of conditional dependence investigated were listed as cases 1 – 6 below. The unadjusted and corrected mean sensitivities and mean specificities of the index test under different variation of conditional dependence between the index test and the reference standard are displayed in Figure 2 (plots a – h). In Figure 2, the Brennerpos represents estimates obtained from the second pair of estimators proposed by Brenner[3], which is employed to correct for the sensitivity and specificity of the index test given that the index test and reference standard are positively correlated. Each plot displayed in Figure 2 labelled a – h is:

a. **Case 1**: The unadjusted sensitivities and specificities of the index test when the index test and reference standard are conditionally dependent among the diseased and non-diseased groups ($cov_d = cov_{nd} = 0.08$).
b. **Case 2**: The unadjusted sensitivities and specificities of the index test when the index test and reference standard are conditionally dependent among the diseased and non-diseased groups ($cov_d = -0.02;\ cov_{nd} = -0.01$).
c. **Case 3**: The unadjusted sensitivities and specificities of the index test when the index test and reference standard are conditionally dependent among the diseased and non-diseased groups ($cov_d = 0.07;\ cov_{nd} = -0.01$).
d. **Case 4**: The unadjusted sensitivities and specificities of the index test when the index test and reference standard are conditionally dependent among the diseased and non-diseased groups ($cov_d = -0.02;\ cov_{nd} = 0.08$).
e. **Case 5a**: The unadjusted sensitivities and specificities of the index test when the index test and reference standard are conditionally dependent among the diseased group ($cov_d = 0.08;\ cov_{nd} = 0$).
f. **Case 5b**: The unadjusted sensitivities and specificities of the index test when the index test and reference standard are conditionally dependent among the diseased group ($cov_d = -0.02;\ cov_{nd} = 0$).
g. **Case 6a**: The unadjusted sensitivities and specificities of the index test when the index test and reference standard are conditionally dependent among the non-diseased group ($cov_d = 0;\ cov_{nd} = 0.08$).
h. **Case 6b**: The unadjusted sensitivities and specificities of the index test when the index test and reference standard are conditionally dependent among the non-diseased group ($cov_d = 0;\ cov_{nd} = -0.01$).

Figure 2: The unadjusted and corrected sensitivities and specificities of the index test under different variations of conditional dependence between the index test and the reference standard

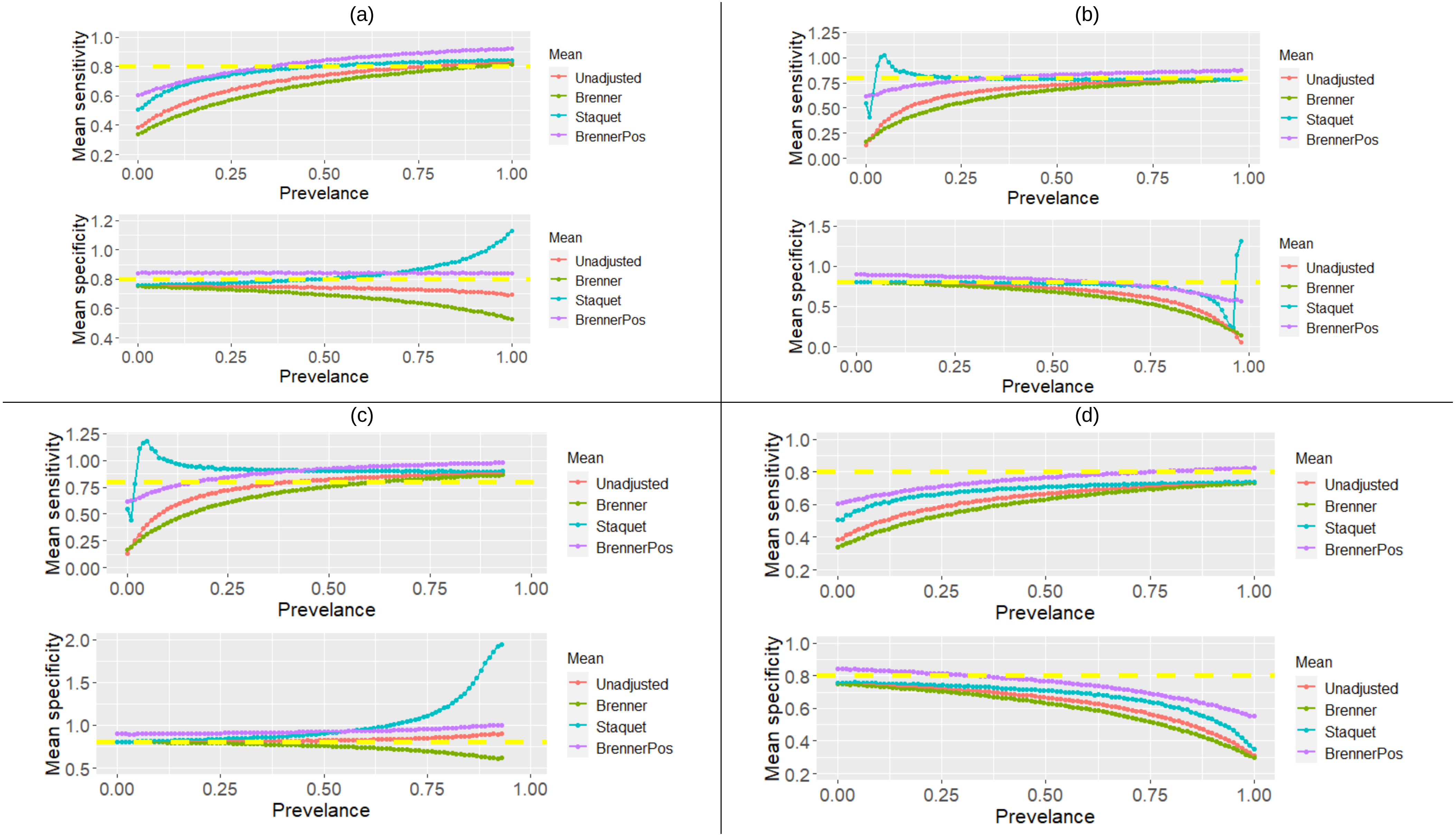

Figure 1 cont.: The unadjusted and corrected sensitivities and specificities of the index test under different variations of conditional dependence between the index test and reference standard

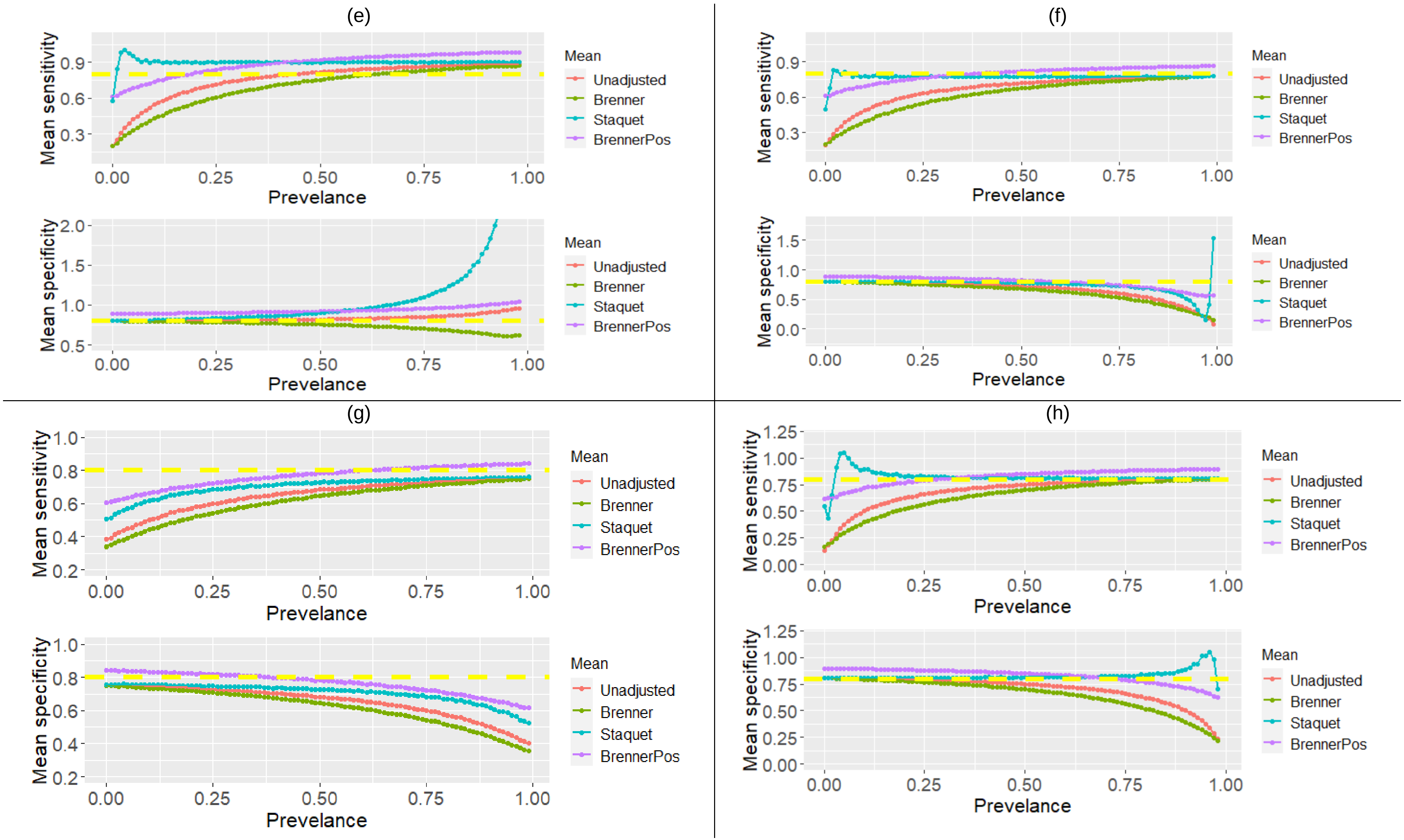

From the plots (2a – 2h), all the methods perform poorly in estimating the sensitivity and specificity as they either over estimated or underestimated the accuracy measures. In addition, the diagnostic accuracy measures estimated from the Staquet et al[4] approach is not constant across different populations unlike when the reference standard and index tests are conditionally independent. Furthermore, there are still illogical results produced when using the Staquet et al[4] methods, especially at very high (> 0.9) or very low (< 0.1) prevalence while the Brenner correction methods and the Classical method do not produce illogical estimates.

In conclusion, when the IT and the RS are conditionally dependent, other statistical method like the Bayesian latent class model should be considered.

References:

1. Wang Z, Dendukuri N, Zar HJ, et al. Modeling conditional dependence among multiple diagnostic tests. *Statistics in medicine* 2017; 36: 4843-4859.
2. Vacek PM. The effect of conditional dependence on the evaluation of diagnostic tests. *Biometrics* 1985; 41: 959-968.
3. Brenner H. Correcting for exposure misclassification using an alloyed gold standard. *Epidemiology* 1996; 7: 406-410. Article.
4. Staquet M, Rozencweig M, Lee YJ, et al. Methodology for the assessment of new dichotomous diagnostic tests. *Journal of Chronic Diseases* 1981; 34: 599-610. Article. DOI: 10.1016/0021-9681(81)90059-X.
